# Supplementary material for: Cancer co-opts differentiation of B-cell precursors into macrophage-like cells
Source: Nat Commun. 2022 Sep 14;13:5376. doi: 10.1038/s41467-022-33117-y (PMC9474882; doi:10.1038/s41467-022-33117-y)
Supplement: Supplementary file 1 — Supplementary Information [file 41467_2022_33117_MOESM1_ESM.pdf]

SUPPLEMENTARY FIGURES

Chen C, et al. "**Cancer coopts differentiation of B-cell precursors into macrophage-like cells**"

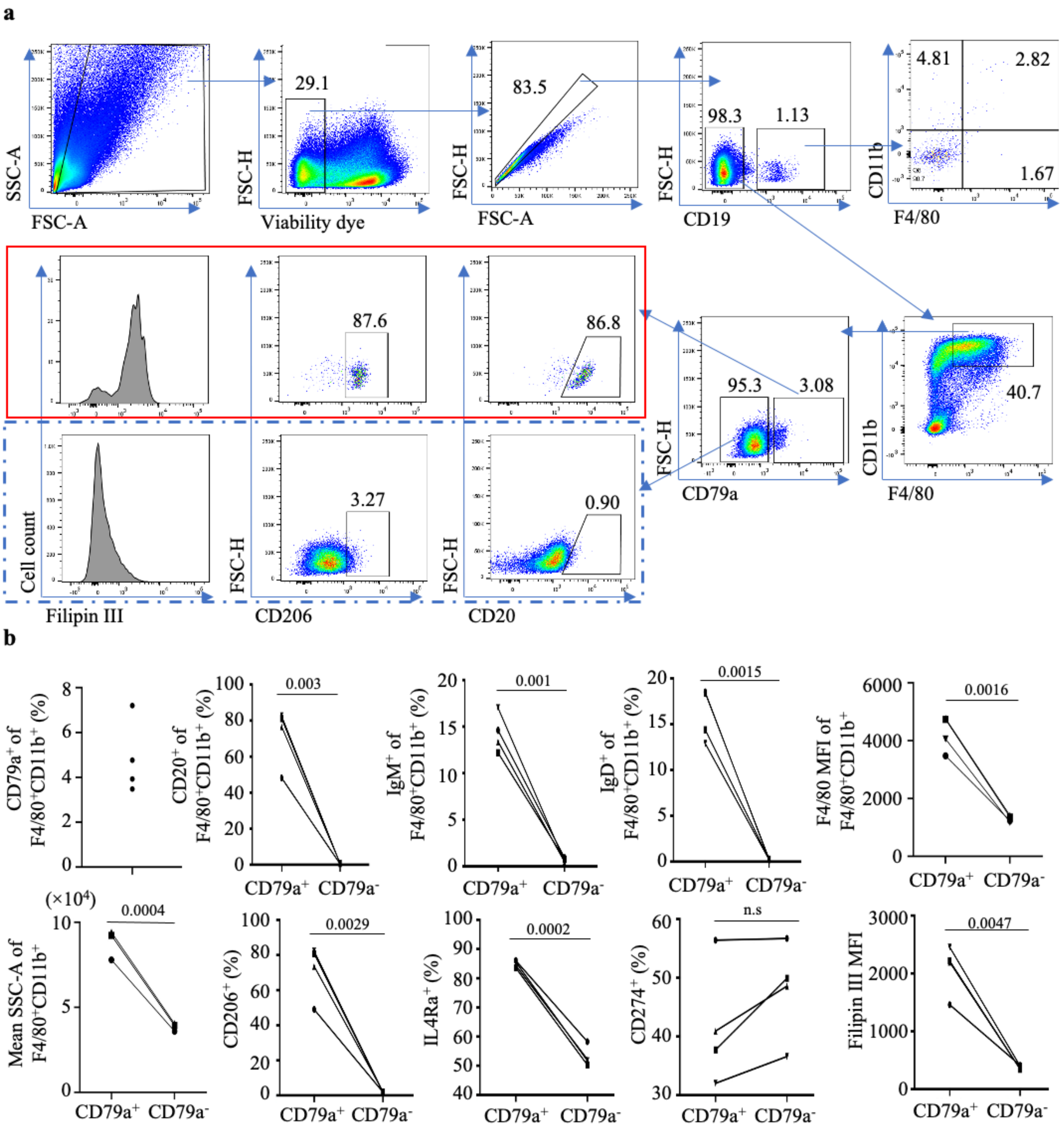

Suppl. Fig.1

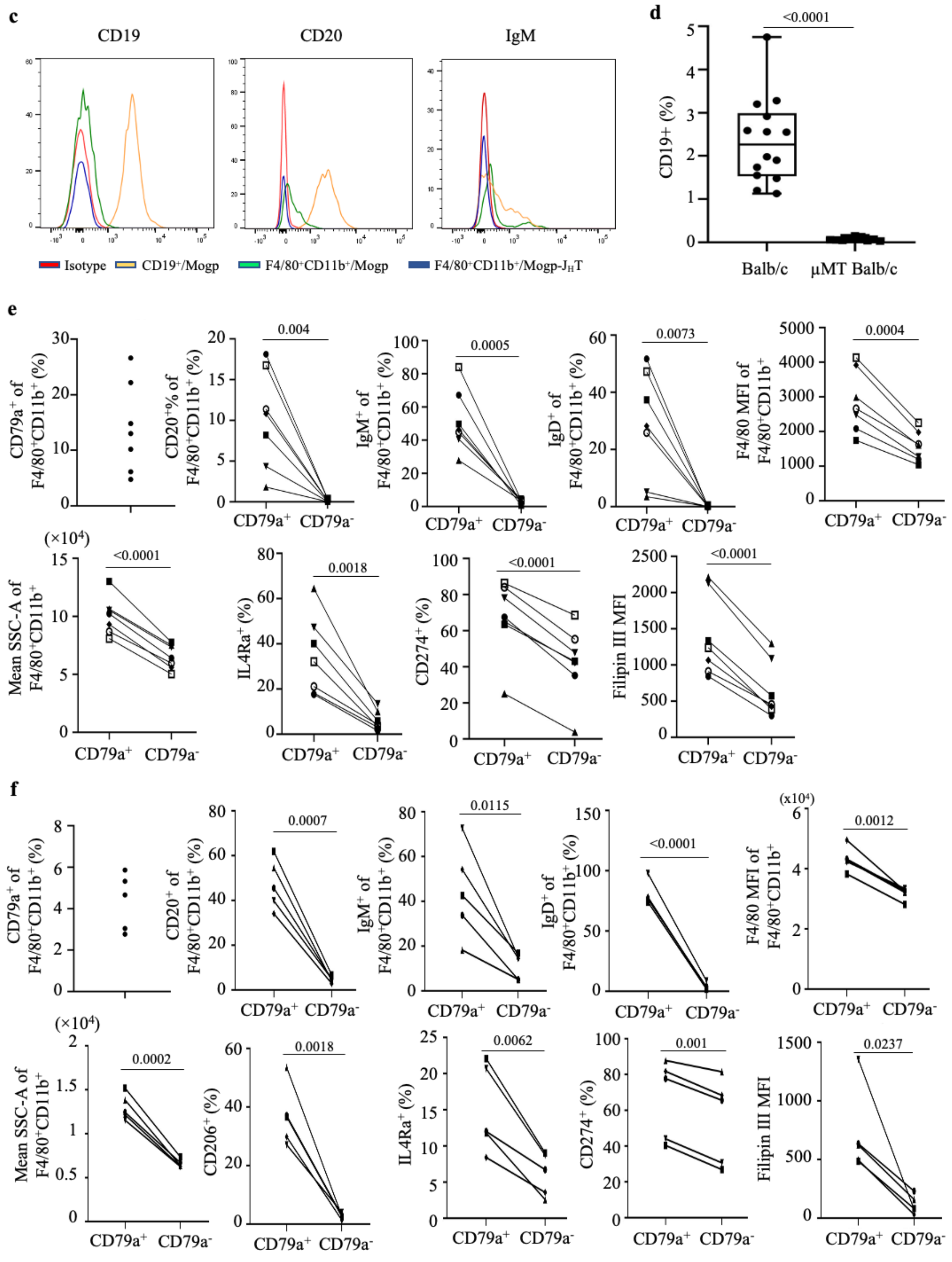
c
CD19
CD20
IgM
Isotype
CD19⁺/Mogp
F4/80⁺CD11b⁺/Mogp
F4/80⁺CD11b⁺/Mogp-JHT
d
<0.0001
CD19+ (%)
Balb/c
μMT Balb/c
e
CD79a⁺ of F4/80⁺CD11b⁺ (%)
CD20⁺% of F4/80⁺CD11b⁺ (%)
0.004
IgM⁺ of F4/80⁺CD11b⁺ (%)
0.0005
IgD⁺ of F4/80⁺CD11b⁺ (%)
0.0073
F4/80 MFI of F4/80⁺CD11b⁺
0.0004
(×10⁴)
Mean SSC-A of F4/80⁺CD11b⁺
<0.0001
IL4Ra⁺ (%)
0.0018
CD274⁺ (%)
<0.0001
Filipin III MFI
<0.0001
CD79a⁺
CD79a⁻
f
CD79a⁺ of F4/80⁺CD11b⁺ (%)
CD20⁺ of F4/80⁺CD11b⁺ (%)
0.0007
IgM⁺ of F4/80⁺CD11b⁺ (%)
0.0115
IgD⁺ of F4/80⁺CD11b⁺ (%)
<0.0001
(x10⁴)
F4/80 MFI of F4/80⁺CD11b⁺
0.0012
(×10⁴)
Mean SSC-A of F4/80⁺CD11b⁺
0.0002
CD206⁺ (%)
0.0018
IL4Ra⁺ (%)
0.0062
CD274⁺ (%)
0.001
Filipin III MFI
0.0237

Suppl. Fig.1

g

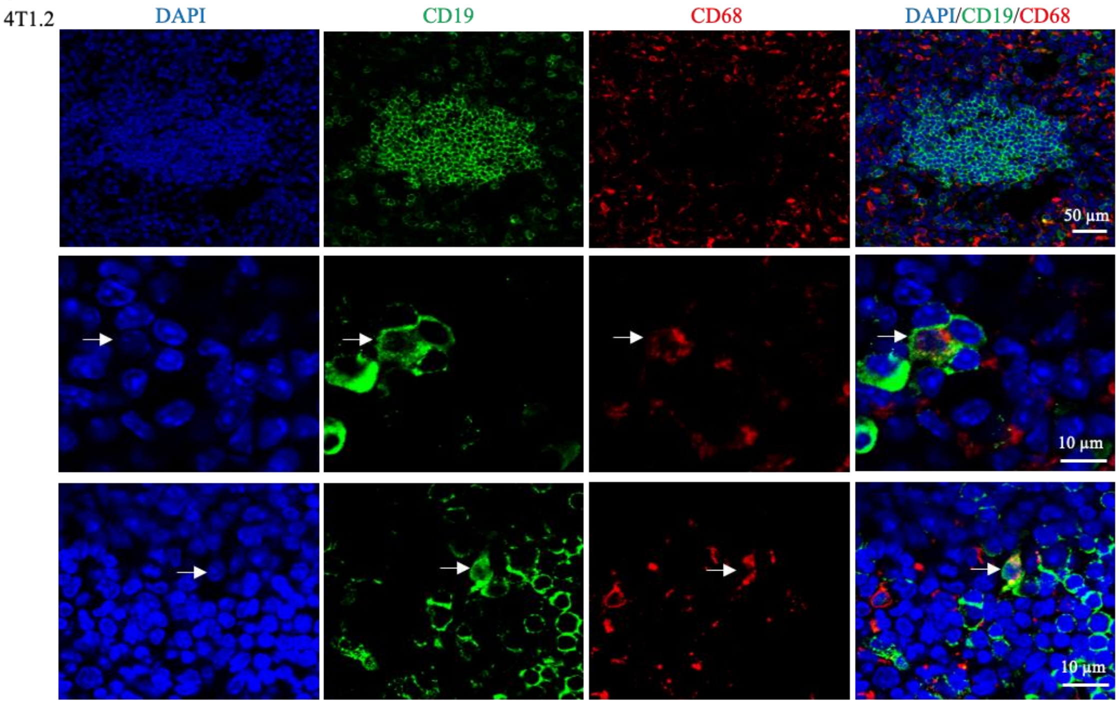

h

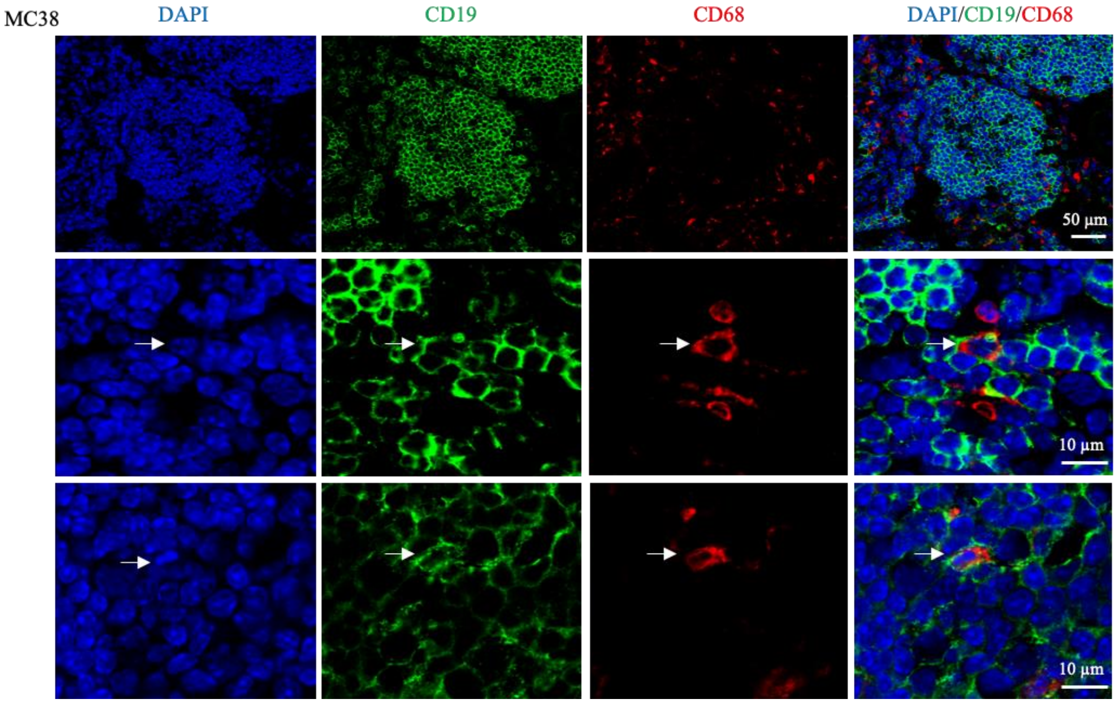

Supplementary Figure 1. Co-expression of B-cell and macrophage specific markers. a, gating strategy showing co-expression of B-cell specific markers (CD19, CD79a, CD20) and macrophage markers (F4/80 and CD11b) in TIB and TAM in the tumor from BALB/c mice with s.c. 4T1.2 cancer. **b,** Representative FACS profiling data of surface markers in CD79a^{+}

TAM (presumably B-MF) as compared with $CD79a^-$ TAM ("classic" TAM) in in the tumor of mice with 4T1.2 breast cancer (n=4). ($CD20^+$ *P*=0.0003, IgM^+ *P*=0.0001, IgD^+ *P*=0.0015, F4/80 MFI *P*=0.0016, Mean SSC-A *P*=0.0004, $CD206^+$ *P*=0.0029, $IL4Ra^+$ *P*=0.0002, Filipin III MFI *P*=0.0047 in $CD79a^+$ vs $CD79a^-$). *P* values were calculated using two-tailed paired *t* test. **c**, Representative histogram showing B-cell specific makers (CD19, CD20 and IgM) within TAM ($F4/80^+CD11b^+$) in the peritoneum of B-cell sufficient (Mogp) and B-cell deficient (Mogp-J_HT) C57BL/6 mice with peritoneal Mogp ovarian cancer. **d**, Box-Whiskers plot quantification of tumor infiltrated $CD19^+$ TIB frequency from B-cell sufficient (BALB/c) vs B-cell deficient (µMT BALB/c) mice with 4T1.2 cancer (n=14 for BALB/c group, n=9 for µMT BALB/c group, *P*<0.0001). *P* values were calculated using two-tailed unpaired *t* test. Representative FACS profiling data of surface markers in $CD79a^+$ TAM as compared with $CD79a^-$ TAM in Mogp mice (n=7, $CD20^+$ *P*=0.0004, IgM^+ *P*=0.0005, IgD^+ *P*=0.0073, F4/80 MFI *P*=0.0004, Mean SSC-A *P*<0.0001, $IL4Ra^+$ *P*=0.0018, $CD274^+$ *P*<0.0001, Filipin III MFI *P*<0.0001 in $CD79a^+$ vs $CD79a^-$, **e**) and C57BL/6 mice with MC-38 cancers (n=5, $CD20^+$ *P*=0.0007, IgM^+ *P*=0.0115, IgD^+ *P*<0.0001, F4/80 MFI *P*=0.0012, Mean SSC-A *P*=0.0002, $CD206^+$ *P*=0.0018, $IL4Ra^+$ *P*=0.0062, $CD274^+$ *P*=0.0001, Filipin III MFI *P*=0.0237 in $CD79a^+$ vs $CD79a^-$, **f**). *P* values were calculated using two-tailed paired *t* test. Representative immunohistochemistry staining for B cells (CD19) and macrophages (CD68) in the tumor of BALB/c and C57BL/6 mice with s.c. 4T1.2 cancer (**g**) and MC38 colon cancer (**h**), respectively. White arrow shows $CD19^+CD68^+$ cells, and scale bars represent 50 µm (upper panel) and 10 µm (lower 2 panels). G and H, n=3 mice per group. B, E and F, each dot is an independent mouse. Results shown here were reproduced at least twice. From here on, Error bars are for SEM.

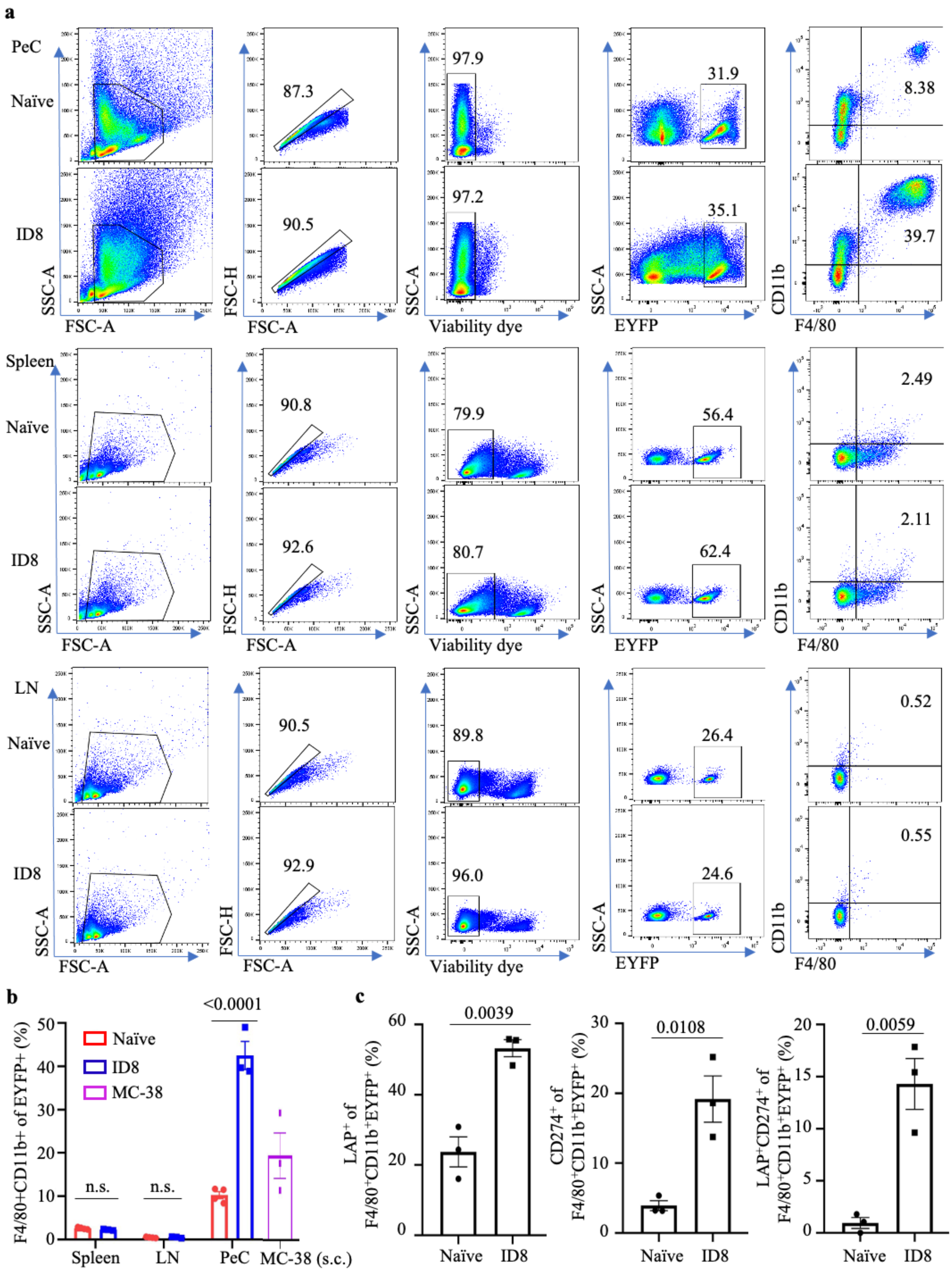

Supplementary Figure 2. EYFP$^+$F4/80$^+$CD11b$^+$ cells in the tumor of naïve and tumor bearing Mb1-EYFP mice. Representative FACS plots (**a**) and quantification (**b**) showing

Mean ± SEM of F4/80$^+$CD11b$^+$ of EYFP$^+$ cells in spleen, LN and PeC (P<0.0001) of Mb1-EYFP mice vs without (naïve) peritoneal ID8 cancer. "MC-38 (s.c.)" is for F4/80$^+$CD11b$^+$ EYFP$^+$ cells (Mean ± SEM) in the tumor of Mb1-EYFP mice with MC38 tumor. (n=4 for naïve group, n=3 for ID8 group and n=3 for MC-38 group). P values were calculated using two-tailed unpaired t test. **c**, Quantification of LAP/TGFβ and PD-L1 expression in F4/80$^+$CD11b$^+$EYFP$^+$ cells (Mean ± SEM) in the peritoneum of naïve vs ID8 cancer-bearing mice (LAP$^+$ P=0.0039, CD274$^+$ P=0.0108, and LAP$^+$CD274$^+$ P=0.0059, n=3/group). P values were calculated using two-tailed unpaired t test. Each symbol is an independent mouse.

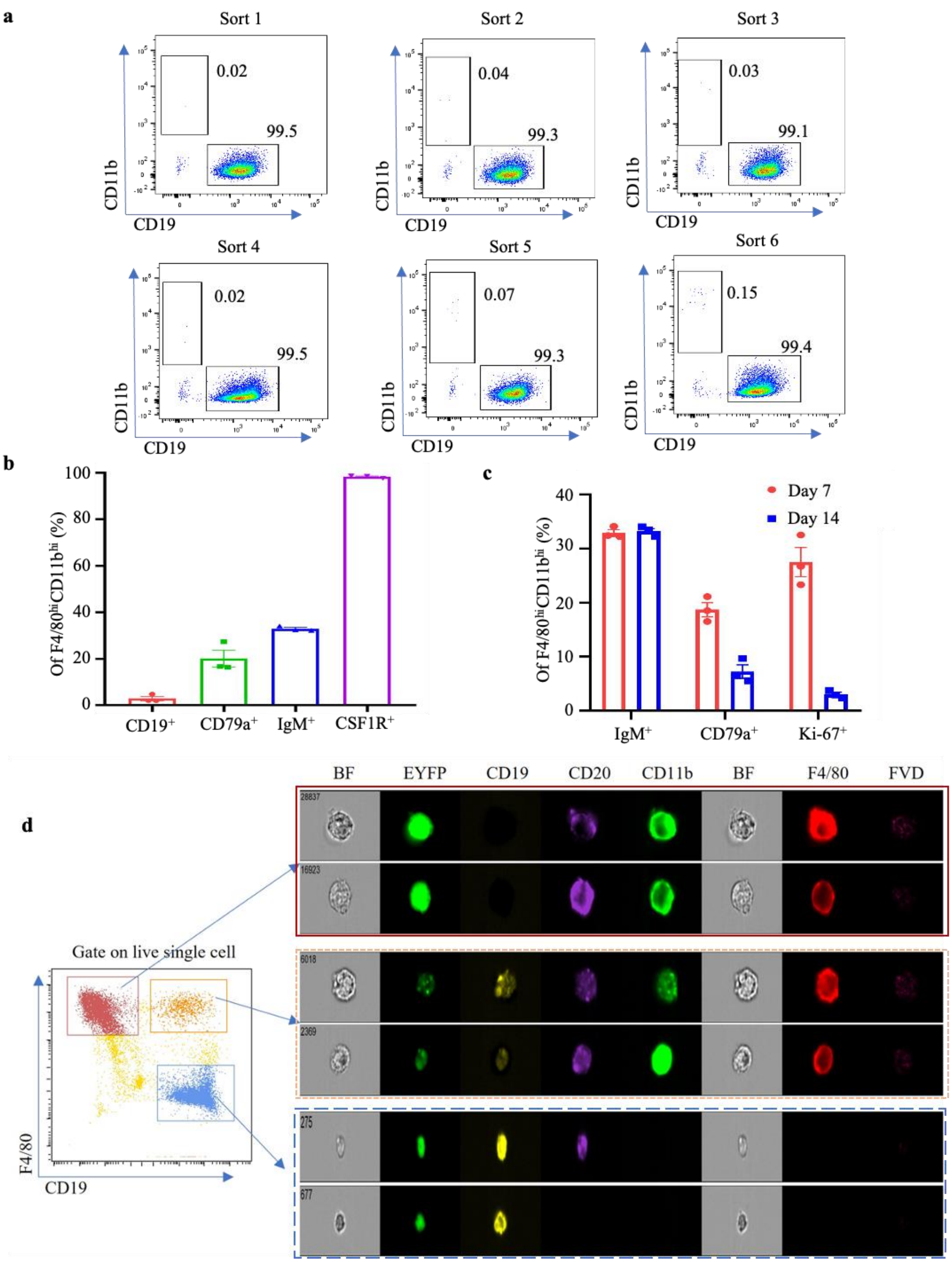
a
Sort 1
0.02
99.5
CD11b
CD19
Sort 2
0.04
99.3
Sort 3
0.03
99.1
Sort 4
0.02
99.5
Sort 5
0.07
99.3
Sort 6
0.15
99.4
b
Of F4/80hiCD11bhi (%)
CD19+
CD79a+
IgM+
CSF1R+
c
Day 7
Day 14
Ki-67+
d
BF
EYFP
CD19
CD20
CD11b
F4/80
FVD
Gate on live single cell
F4/80

Suppl. Fig.3

e

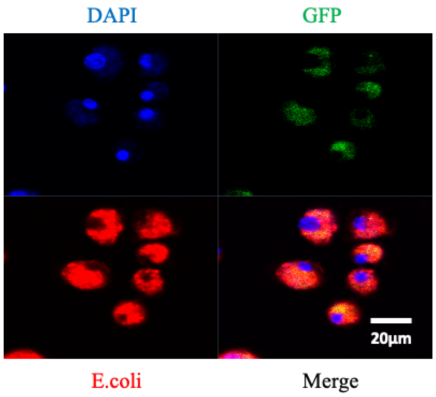

f

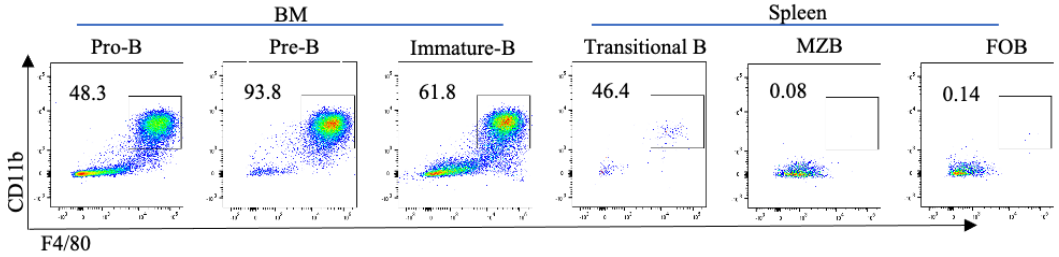

g

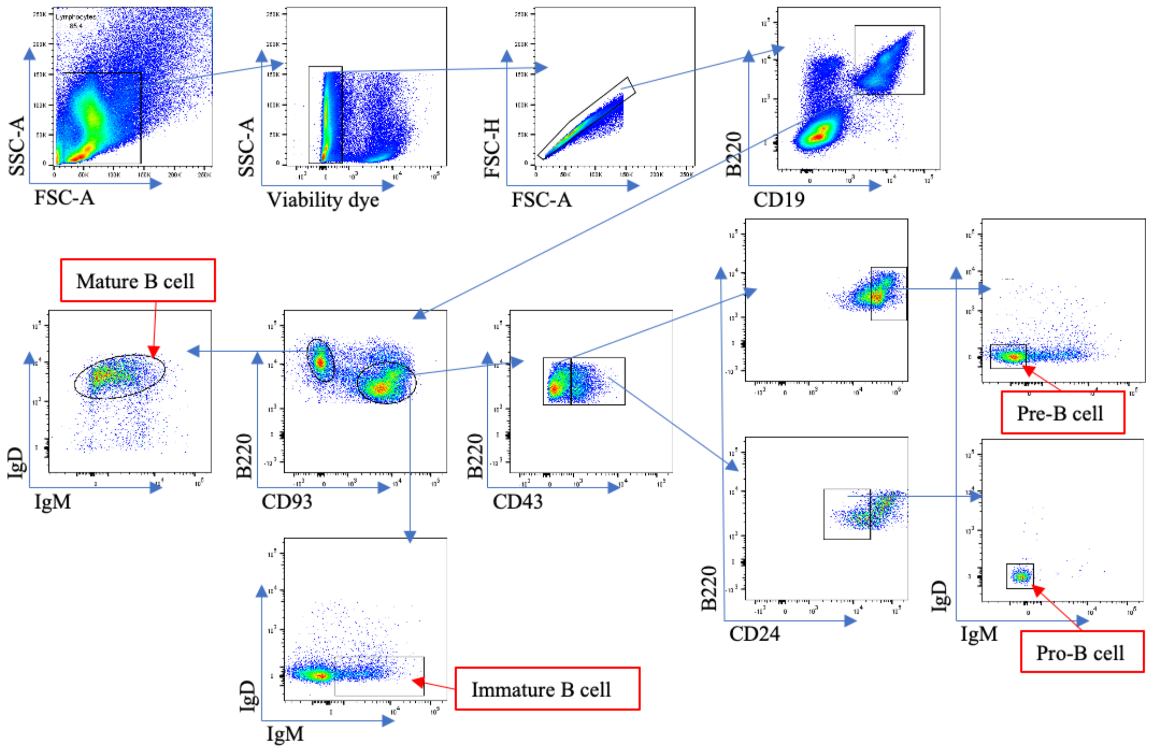

Suppl. Fig.3

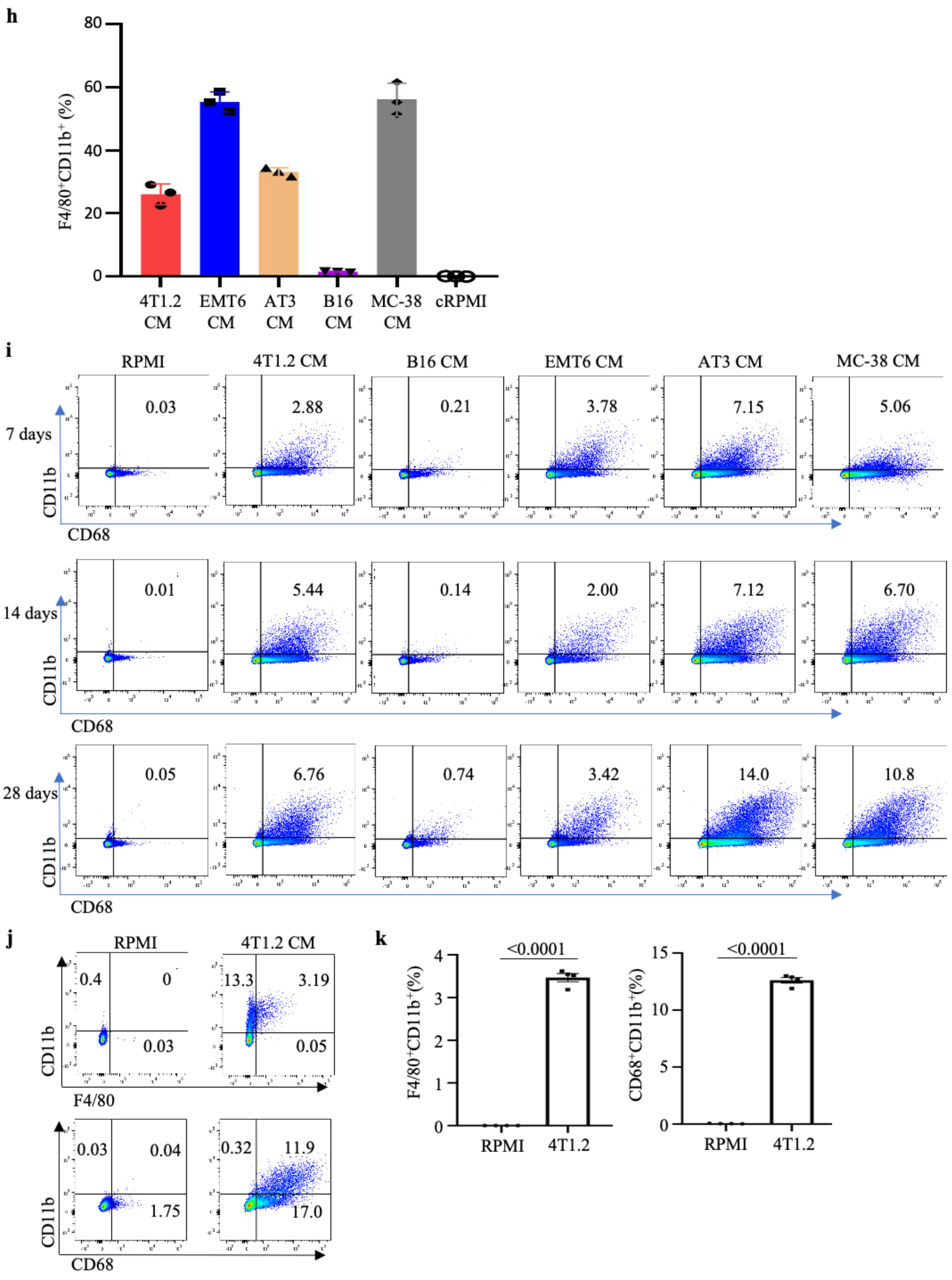

Supplementary Figure 3. Cancer CM causes differentiation of BM B cells into macrophages. a, representative FACS plots showing the purity (> 99%) of $CD19^+$ B cells independently FACS-purified from 6 mice (sort 1-6), which we used in the *in vitro* B-MF

conversion assays. **b** and **c,** Quantification of FACS results (Mean ± SEM) for indicated expression of markers (X-axis) in F4/80$^+$CD11b$^+$ cells generated from BM B cells cultured in 4T1.2 CM for 7 (n=3, **b**) and 14 days (red, 7 days, and blue, 14 days, n=3/group, **c**). **d**, Representative Imagestream images for expression of EYFP, F4/80, CD11b, CD19, and CD20 in single CD19$^+$F4/80$^-$, CD19$^+$F4/80$^+$ and CD19$^-$F4/80$^+$ cells (gated and arrows) after incubation of BM B cells of EYFP$^+$ mice in 4T1.2-CM for 7 days. See that pure B cells (F4/80$^-$CD19$^+$ cells) are smaller in size than F4/80$^+$ cells. **e**, Immunofluorescent microscopy image showing B-MF generated from RAG2-GFP$^+$ BM B-cells can phagocytize pHrodo™ red-labeled *E. coli*. Reproduced twice. Scale bar represents 20 µm. **f,** Representative FACS plots showing FACS-purified BM B-cell precursors (pro-B and pre-B cells) and immature B cells, but not splenic transitional, marginal zone and follicular B cells, can generate F4/80$^+$CD11b$^+$ cells upon culture with 4T1.2-CM for 7 days. **g,** Gating strategy of FACS-purified BM B-cell subsets used. **h**, Quantification of results shown in Fig2G, showing Mean ± SEM of F4/80$^+$CD11b$^+$ cells after culture of BM B cells in cRPMI or CM from 4T1.2, EMT6, AT3, B16-F10, and MC38 cancer cells (n=3 per group). **i**, Representative FACS plots of pre-B cell line 70z/3 cultured for 7, 14, or 28 days with CM from indicated cancer cells. Shows surface upregulation of CD11b and CD68. **j**, Representative FACS plots and **k,** quantification (Mean ± SEM, n=4 per group) for surface expression of macrophage markers in 70z/3 cells after 30-day culture in cRPMI vs 4T1.2-CM. *P* values were calculated using two-tailed unpaired *t* test. The results shown here were independently reproduced at least three times.

a

BM B cell and monocyte sort gating strategy

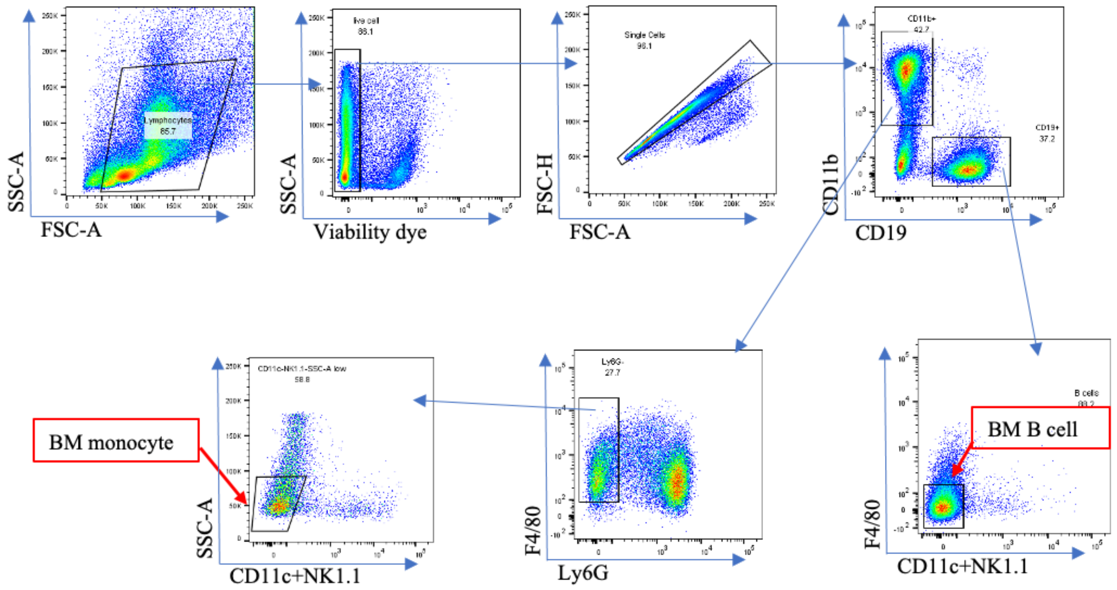

b

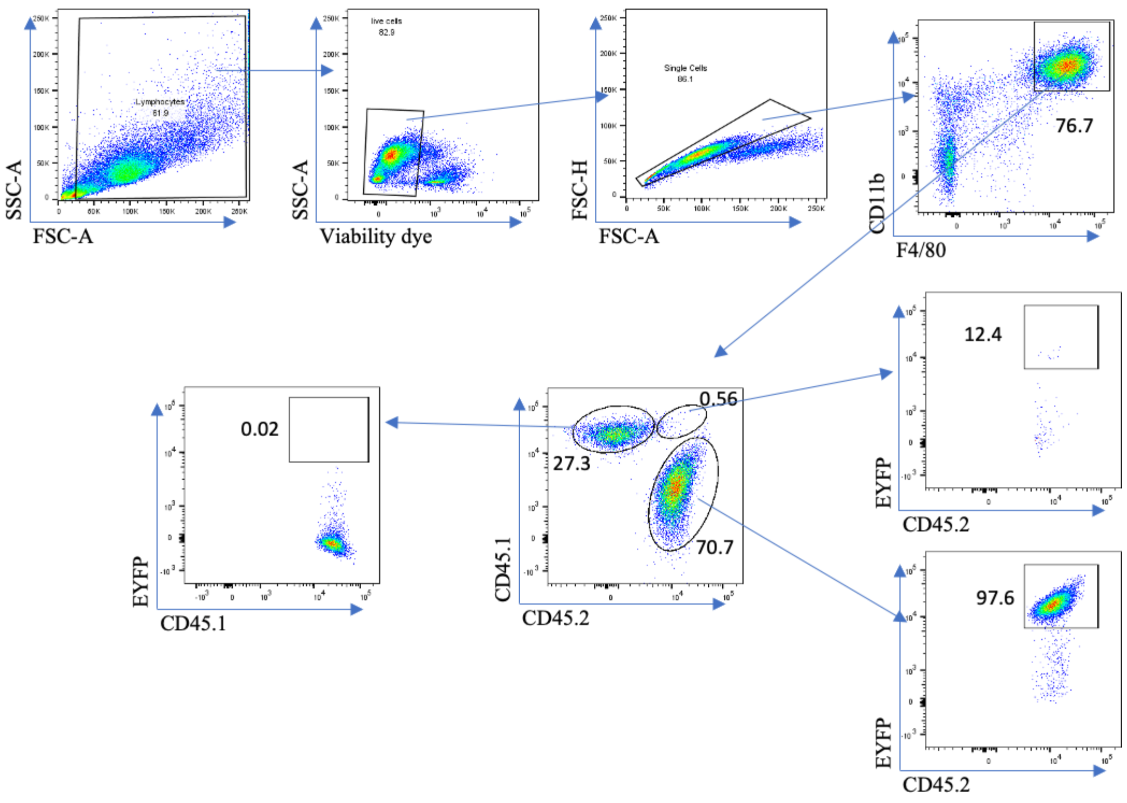

Suppl. Fig.4

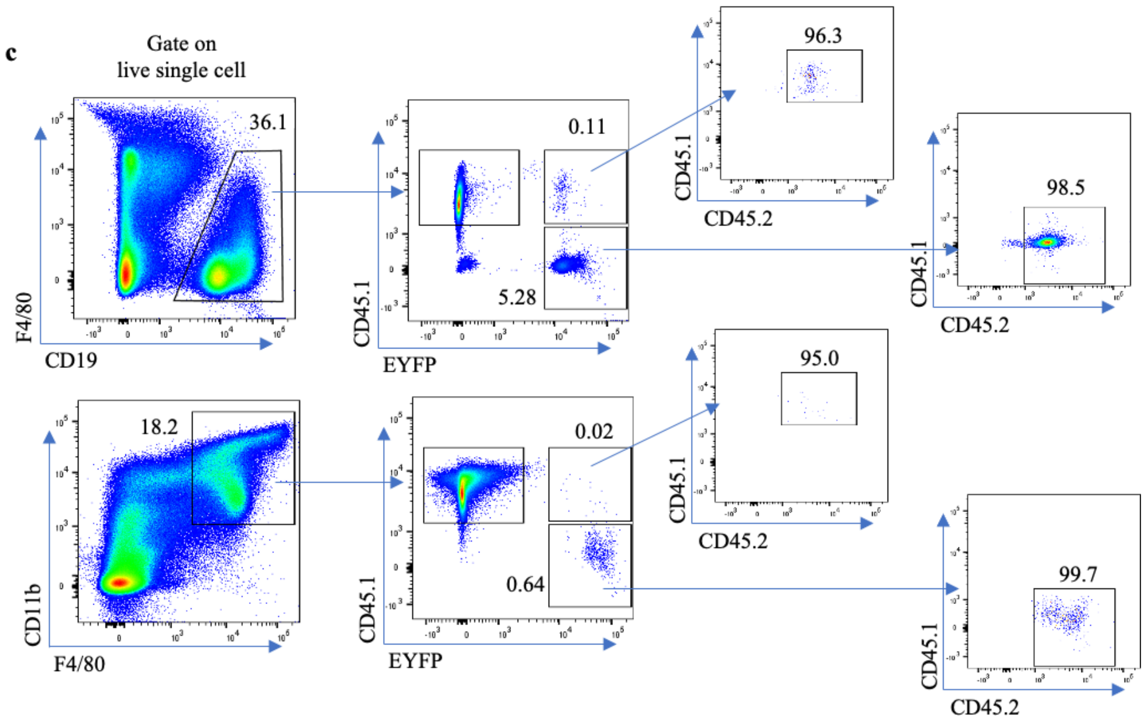

Supplementary Figure 4. Gating strategy of FACS-sorted cells and cells used in vivo in Fig.2h-j. a, Gating strategy of FACS-sorted BM B cells and monocytes. **b**, Gating strategy of the experiment depicted in Fig 2H. It shows CD45.1^{+} and CD45.2^{+} F4/80^{+}CD11b^{+} macrophages after a mixed culture of CD45.1^{+} monocytes and EYFP^{+}CD45.2^{+} BM B cells in 4T1.2-CM. **c**, Gating strategy of the experiment depicted in Fig 2I. It shows EYFP, surface CD45.1 and CD45.2 expression in peritoneal F4/80^{+}CD11b^{+} (macrophages) and CD19^{+} B cells in CD45.1 mice with ID8 cancer. EYFP^{+}CD45.2^{+} BM B cells were i.p. transferred into CD45.1 mice with ID8 cancer in the peritoneum.

a

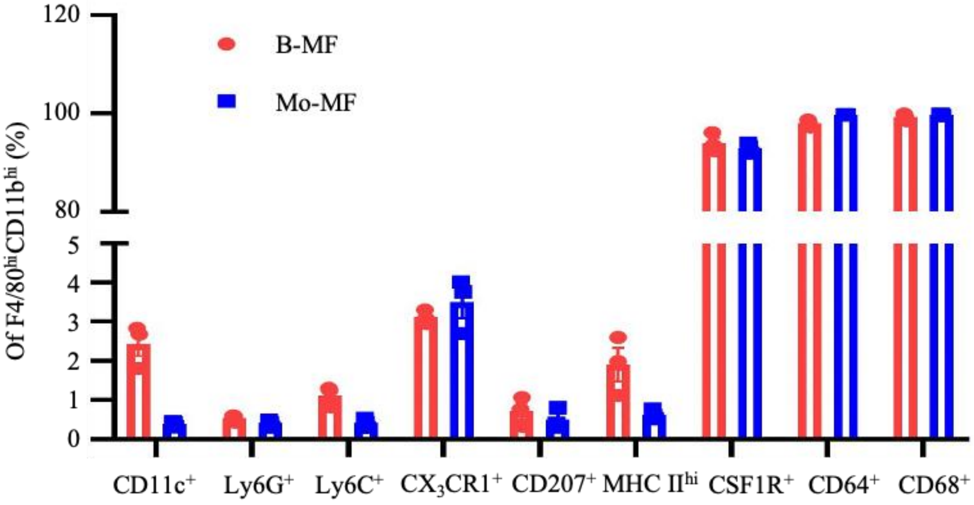

b

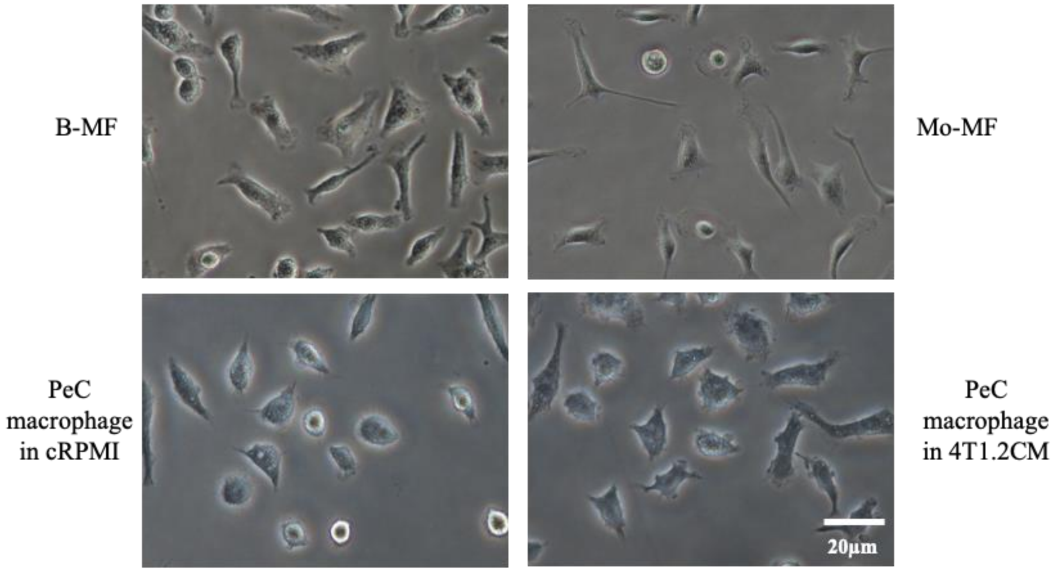

Suppl. Fig.5

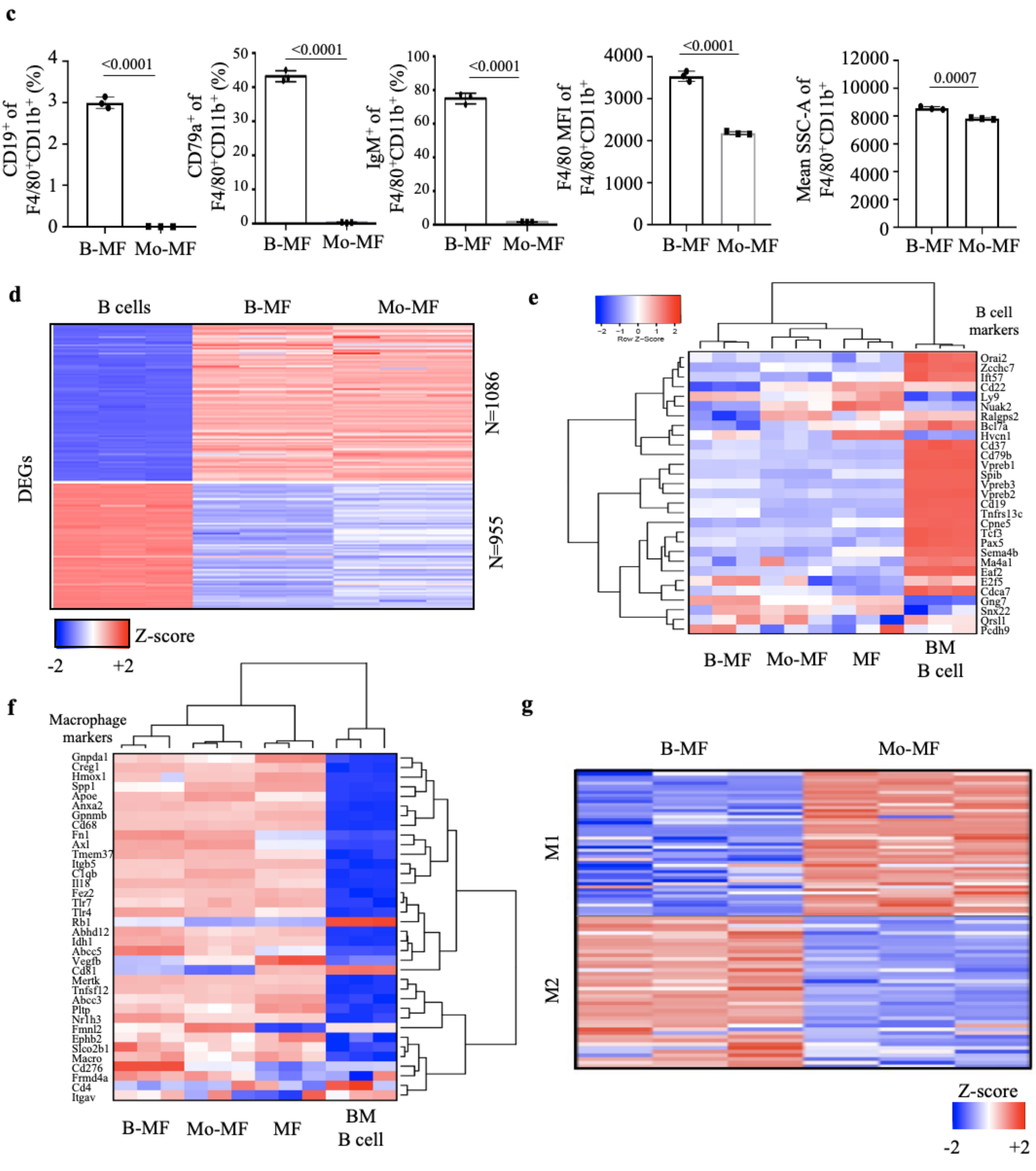
c
<0.0001
CD19+ of F4/80+CD11b+ (%)
B-MF
Mo-MF
<0.0001
CD79a+ of F4/80+CD11b+ (%)
<0.0001
IgM+ of F4/80+CD11b+ (%)
<0.0001
F4/80 MFI of F4/80+CD11b+
0.0007
Mean SSC-A of F4/80+CD11b+
d
B cells
B-MF
Mo-MF
DEGs
N=1086
N=955
Z-score
-2
+2
e
Row Z-Score
B cell markers
Orai2
Zcchc7
Ift57
Cd22
Ly9
Nuak2
Ralgps2
Bcl7a
Hvcn1
Cd37
Cd79b
Vpreb1
Spib
Vpreb3
Vpreb2
Cd19
Tnfrs13c
Cpne5
Tcf3
Pax5
Sema4b
Ms4a1
Eaf2
E2f5
Cdca7
Gng7
Snx22
Qrsl1
Pcdh9
B-MF
Mo-MF
MF
BM B cell
f
Macrophage markers
Gnpda1
Creg1
Hmox1
Spp1
Apoe
Anxa2
Gpnmb
Cd68
Fn1
Axl
Tmem37
Itgb5
C1qb
Il18
Fez2
Tlr7
Tlr4
Rb1
Abhd12
Idh1
Abcc5
Vegfb
Cd81
Mertk
Tnfsf12
Abcc3
Pltp
Nr1h3
Fmnl2
Ephb2
Slco2b1
Macro
Cd276
Frmd4a
Cd4
Itgav
B-MF
Mo-MF
MF
BM B cell
g
B-MF
Mo-MF
M1
M2
Z-score
-2
+2

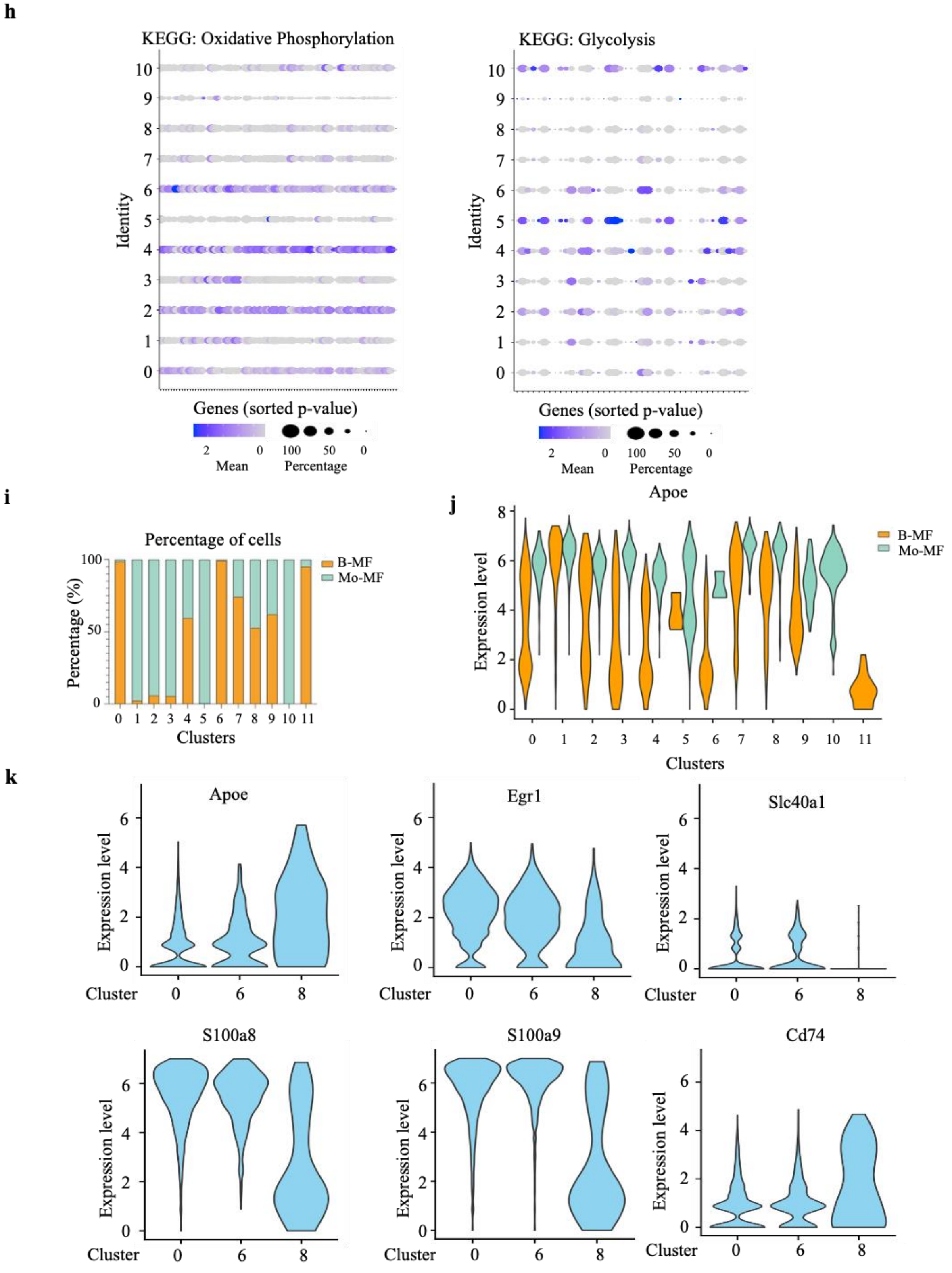

Supplementary Figure 5. Distinct gene expression profiles of B-MF and Mo-MF. a,

Quantification of FACS staining shows Mean ± SEM expression of indicated markers (X-axis) on the surface of *in vitro* generated F4/80^{+}CD11b^{+} B-MF vs Mo-MF (CD19^{+} *P*<0.0001, CD79a^{+} *P*<0.0001, IgM^{+} *P*<0.0001, F4/80 MFI *P*<0.0001, and Mean SSC-A *P*=0.0007, n=3 per group). **b**, Representative light microscopy images showing morphology of *in vitro* generated B-MF and Mo-MF as compared to macrophages from peritoneum (PeC) of naïve mice, which were cultured in cRPMI or 4T1.2-CM. Independently reproduced twice. Scale bar represented 20 µm. **c**, *in vitro* generated B-MF differ from Mo-MF. Shown are frequency Mean ± SEM of CD19, CD79a and IgM expression and MFI Mean ± SEM of F4/80 within F4/80^{+} CD11b^{+} cells and Mean ± SEM of cell sizes (n=3 per group). *P* values were calculated using two-tailed unpaired *t* test between B-MF vs Mo-MF. **d**, Heatmap showing 2041 differential expressed genes between B cells and macrophages (B-MF and Mo-MF) identified from mRNA expression profiling (microarray). Scale bar is for expression z-score. Heatmap of expression of canonical B-cell (**e**) and macrophage (**f**) genes in B-MF, Mo-MF, peritoneal macrophages (MF) and BM B cells. **g,** Heatmap showing expression of M1and M2 signature genes (M1/M2 signature genes were from GSE5099) in B-MF and Mo-MF. **h**, KEGG oxidative phosphorylation and glycolysis pathway gene signature in in vitro generated B-MF and Mo-MF in scRNA-seq data. **i,** Frequency of B-MF or Mo-MF in the individual cell clusters **j,** Violin plot of *Apoe* expression in the B-MF and Mo-MF among 12 clusters. **k,** Violin plot of expression of *Apoe, Egr1, Slc40a1, S100a8, S100a9* and *CD74* in clusters 0, 6, 8 identified from scRNA-seq results of TAM from mice with 4T1.2 cancer.

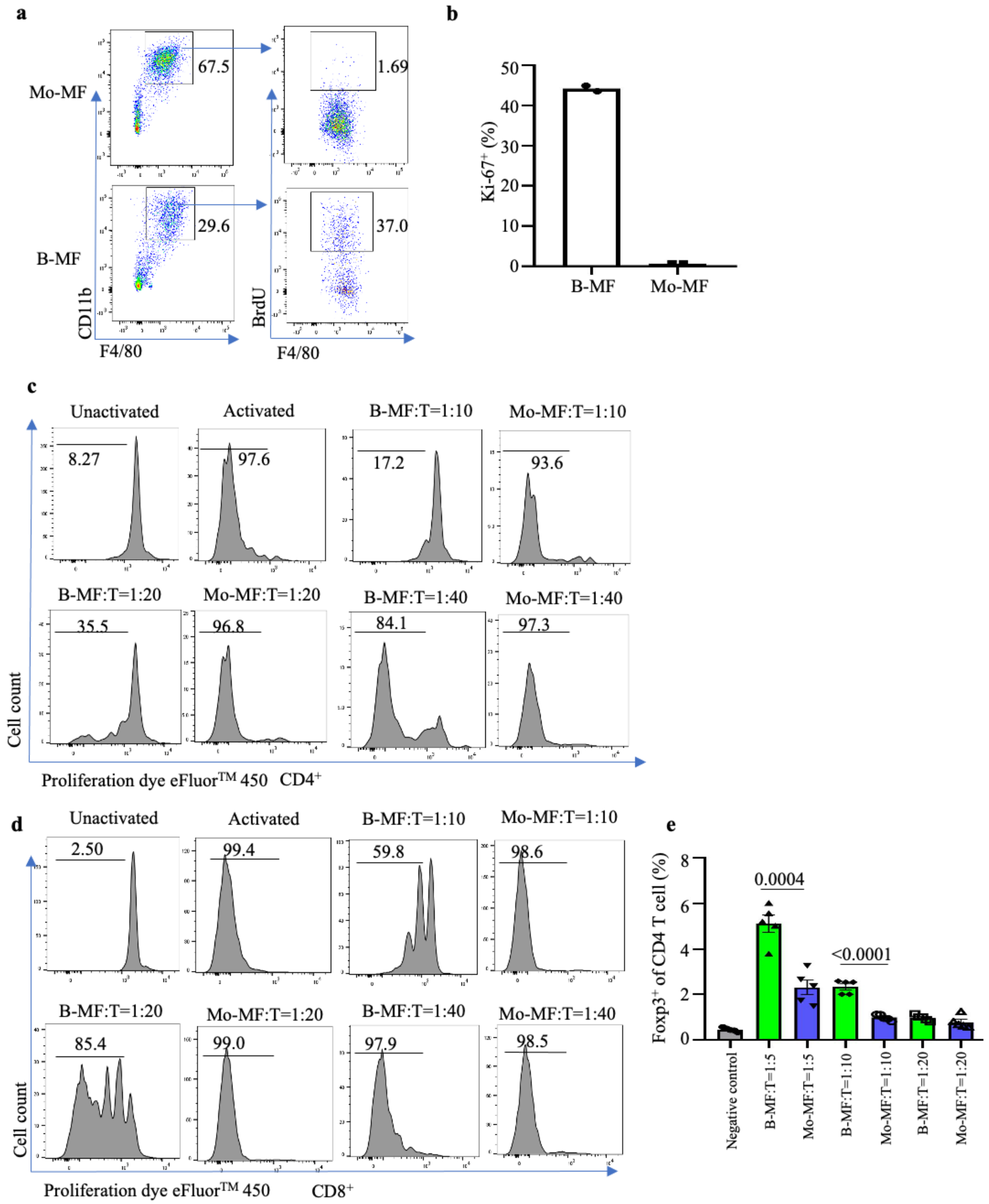
a
Mo-MF
B-MF
67.5
1.69
29.6
37.0
CD11b
F4/80
BrdU
F4/80
b
Ki-67+ (%)
B-MF
Mo-MF
c
Unactivated
Activated
B-MF:T=1:10
Mo-MF:T=1:10
8.27
97.6
17.2
93.6
B-MF:T=1:20
Mo-MF:T=1:20
B-MF:T=1:40
Mo-MF:T=1:40
35.5
96.8
84.1
97.3
Cell count
Proliferation dye eFluorTM 450 CD4+
d
Unactivated
Activated
B-MF:T=1:10
Mo-MF:T=1:10
2.50
99.4
59.8
98.6
B-MF:T=1:20
Mo-MF:T=1:20
B-MF:T=1:40
Mo-MF:T=1:40
85.4
99.0
97.9
98.5
Cell count
Proliferation dye eFluorTM 450 CD8+
e
Foxp3+ of CD4 T cell (%)
0.0004
<0.0001
Negative control
B-MF:T=1:5
Mo-MF:T=1:5
B-MF:T=1:10
Mo-MF:T=1:10
B-MF:T=1:20
Mo-MF:T=1:20

Suppl.Fig.6

f

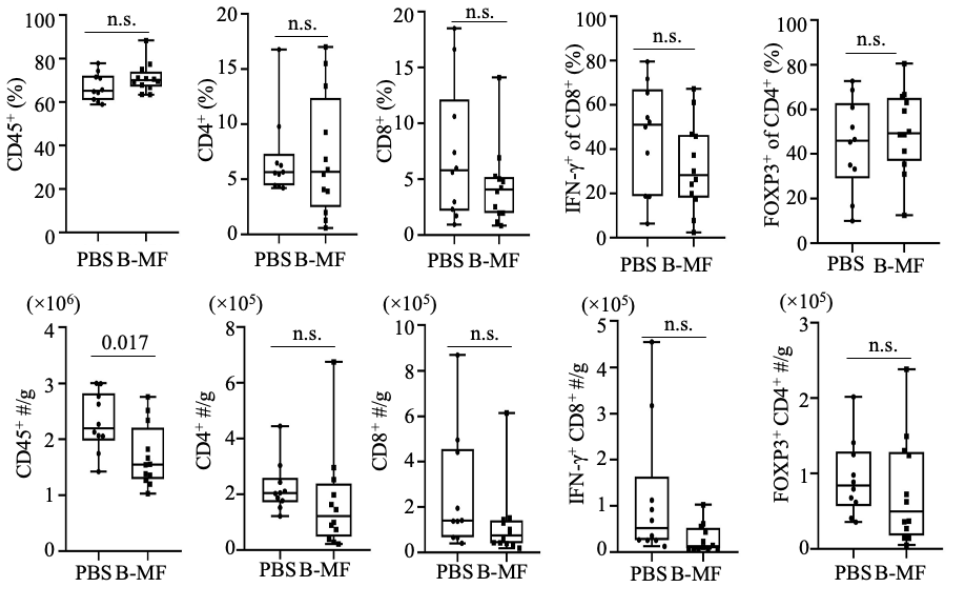

g

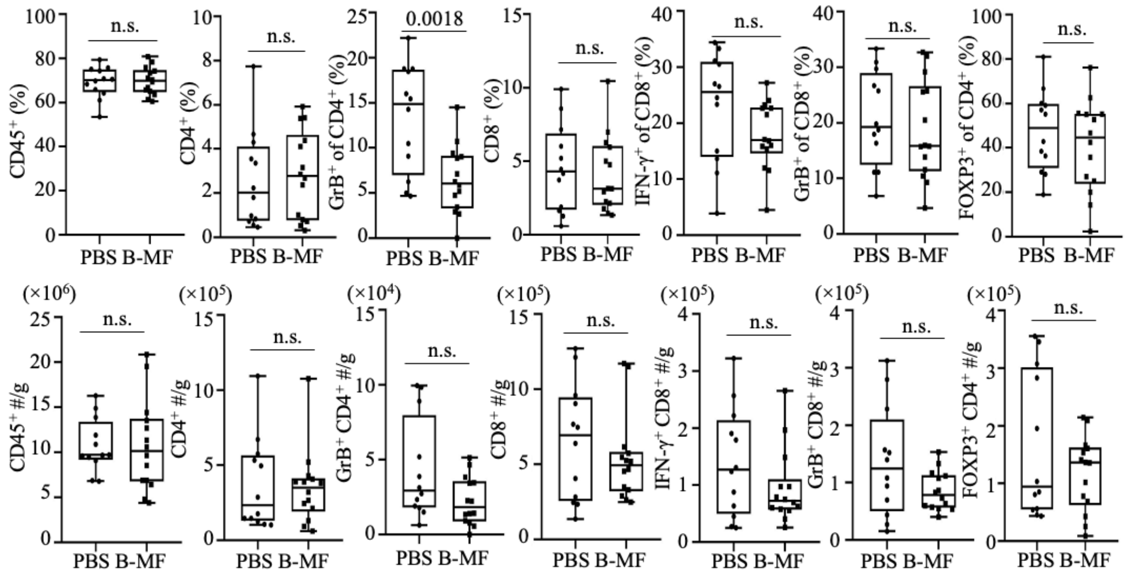

Suppl. Fig.6

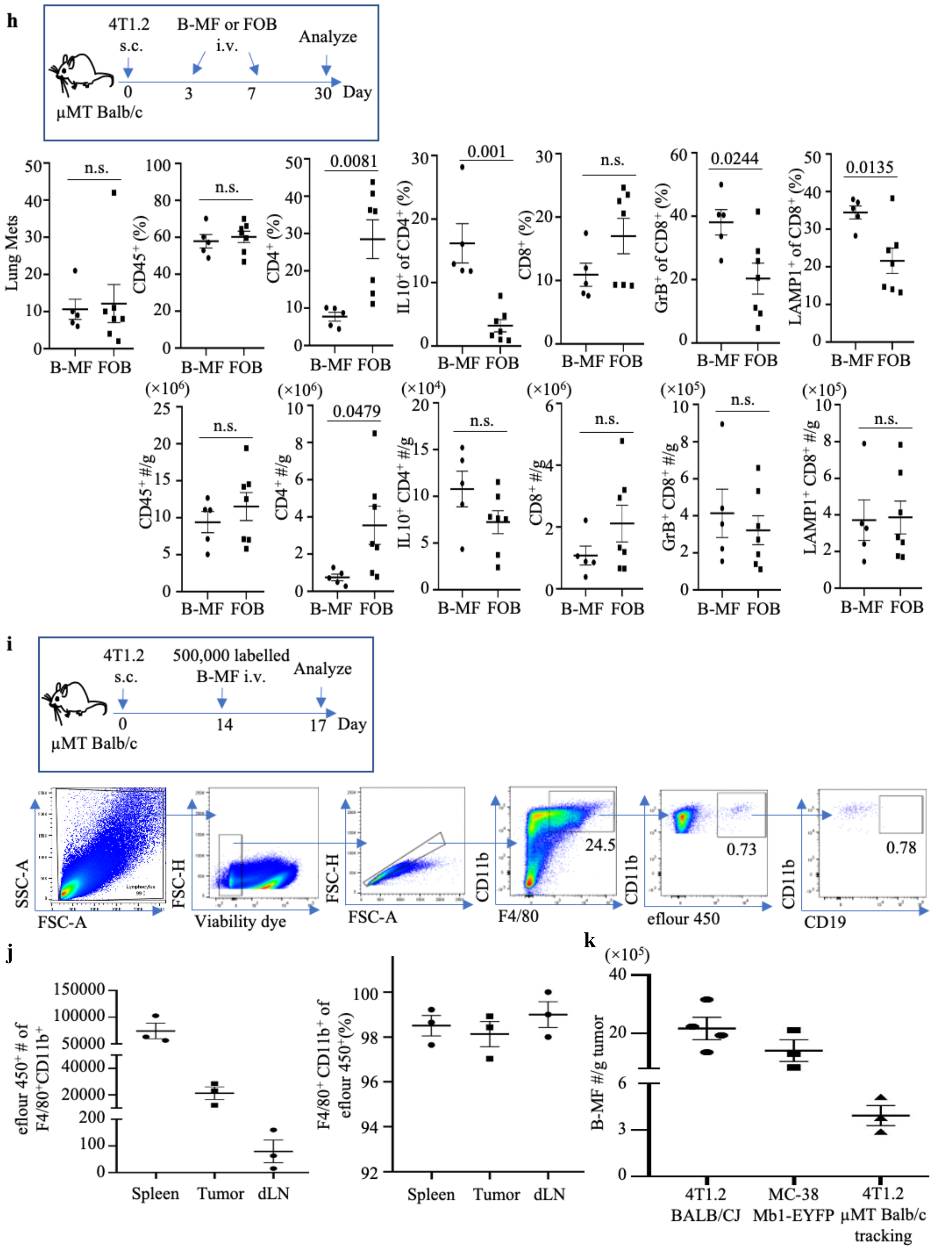

Supplementary Figure 6. B-MF and Mo-MF are functionally different cells. a, Representative FACS plots showing BrdU^{+} frequency in *in vitro* generated B-MF and Mo-MF after pulsing with BrdU for 24h. **b**, Quantification of Ki-67 expression of *in vitro* generated B-MF and Mo-MF, n=2. **c** and **d**, Unlike Mo-MF, B-MF suppress proliferation of T cells *in vitro*. Representative histogram showing the proliferation of CD4^{+} T cells (**c**)

and $CD8^+$ T cells (**d**) after B-MF and Mo-MF were cultured with T cells stimulated with anti-CD3/CD28 Abs at 10:1, 20:1 and 40:1 (T cell: MF) ratio for 4 days. **e,** Quantification (Mean ± SEM, n=5 per group) of $FoxP3^+$ Tregs *in vitro*-converted from $CD25^-CD4^+$ non-Tregs T cells (isolated from naïve mouse spleen) after 5-day co-culture with B-MF vs Mo-MF at 5:1(P=0.0004), 10:1(P<0.0001) and 20:1 (T cell: MF) ratio. P values were calculated using two-tailed unpaired t test. **f** and **g**, Box-Whiskers plot quantification (frequency and absolute numbers) of tumor-infiltrating $CD4^+$ T cells and $CD8^+$ T cells within $CD45^+$ cells and their GrB and IFN-γ -expressing cells, and $FoxP3^+CD4^+$ T cells in s.c. tumor of μMT C57BL/6 mice with B16-F10 melanoma (n=10 for PBS group, n=12 for B-MF group, $CD45^+$#/g P=0.017, **f**) vs PBS and 4T1.2 μMT BALB/c mice (n=12 for PBS group, n=14 for B-MF group, GrB^+% of $CD4^+$ P=0.0018, **g**) vs PBS. P values were calculated using two-tailed unpaired t test. Schema of adoptive transfer experiment in μMT BALB/c mice (upper panel, **h**). Equal numbers of in vitro-generated B-MF or FACS-purified FOB ($3x10^5$ cells/mouse) were i.v. injected 3 and 10 days after s.c. challenge with 4T1.2. cancer (n=5 for B-MF group, n=8 for FOB group). Lung metastasis and tumor-infiltrated cells (TIL) were quantified at day 30 (lower panel, **h**). Shown are frequency within $CD45^+$ cells and absolute numbers of TIL ($CD4^+$% P=0.0081, $IL10^+$% of $CD4^+$ P=0.001, GrB^+% of $CD8^+$ P=0.0244, $LAMP1^+$% of $CD8^+$ P=0.0135, $CD4^+$#/g P=0.0479, **h**) after B-MF vs FOB. P values were calculated using two-tailed unpaired t test. Schema of B-MF tracking experiment (**i**). eflour450-labelled B-MF ($5x10^5$) were i.v. injected into μMT BALB/c mice bearing a 14-day 4T1.2 tumor. After 3 days, absolute numbers (Mean ± SEM, n=3 per group) of $eFluor450^+$ in $CD11b^+F4/80^+$ were quantified (left panel, **j**) and proportion of $CD11b^+F4/80^+$ in $eFluor450^+$ cells were determined by FACS (right panel, **j**) in indicated tissues and in the tumor. Absolute numbers of transferred $eFluor450^+$ $CD11b^+F4/80^+$ cells per gram tumor was compared with that of B-MF in 4T1.2 tumor in BALB/c mice ($CD79a^+F4/80^+CD11b^+CD19^-$) and in MC38 tumor in Mb1-EYFP mice ($EYFP^+F4/80^+CD11b^+CD19^-$, **k**). Each symbol in **b** and **e**-**j**) is for independent mouse.

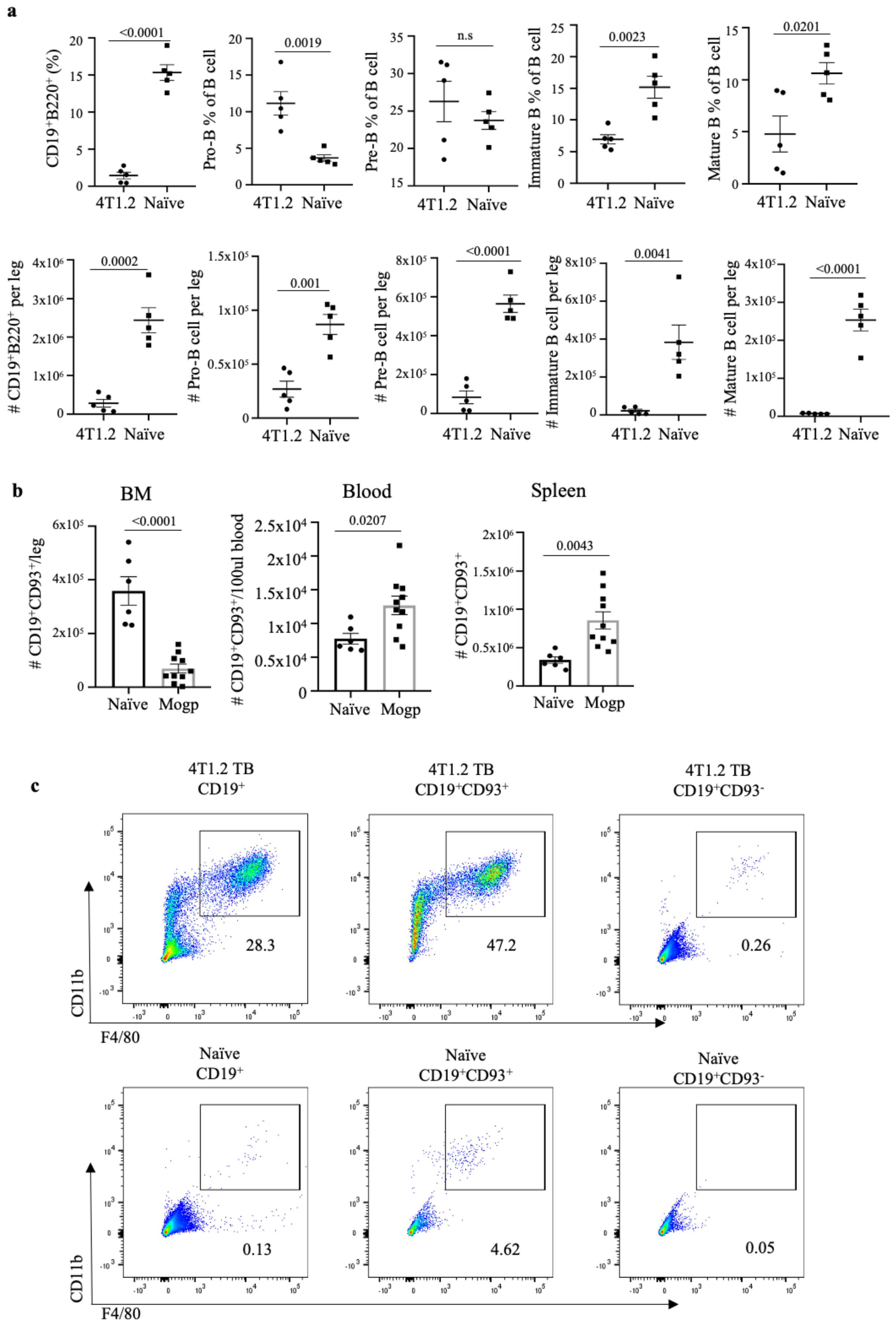
a
CD19⁺B220⁺ (%)
<0.0001
4T1.2 Naïve
Pro-B % of B cell
0.0019
Pre-B % of B cell
n.s
Immature B % of B cell
0.0023
Mature B % of B cell
0.0201
CD19⁺B220⁺ per leg
0.0002
Pro-B cell per leg
0.001
Pre-B cell per leg
<0.0001
Immature B cell per leg
0.0041
Mature B cell per leg
<0.0001
b
BM
Blood
Spleen
CD19⁺CD93⁺/leg
CD19⁺CD93⁺/100ul blood
CD19⁺CD93⁺
0.0207
0.0043
Naïve Mogp
c
4T1.2 TB CD19⁺
4T1.2 TB CD19⁺CD93⁺
4T1.2 TB CD19⁺CD93⁻
28.3
47.2
0.26
Naïve CD19⁺
Naïve CD19⁺CD93⁺
Naïve CD19⁺CD93⁻
0.13
4.62
0.05
CD11b
F4/80

Suppl. Fig.7

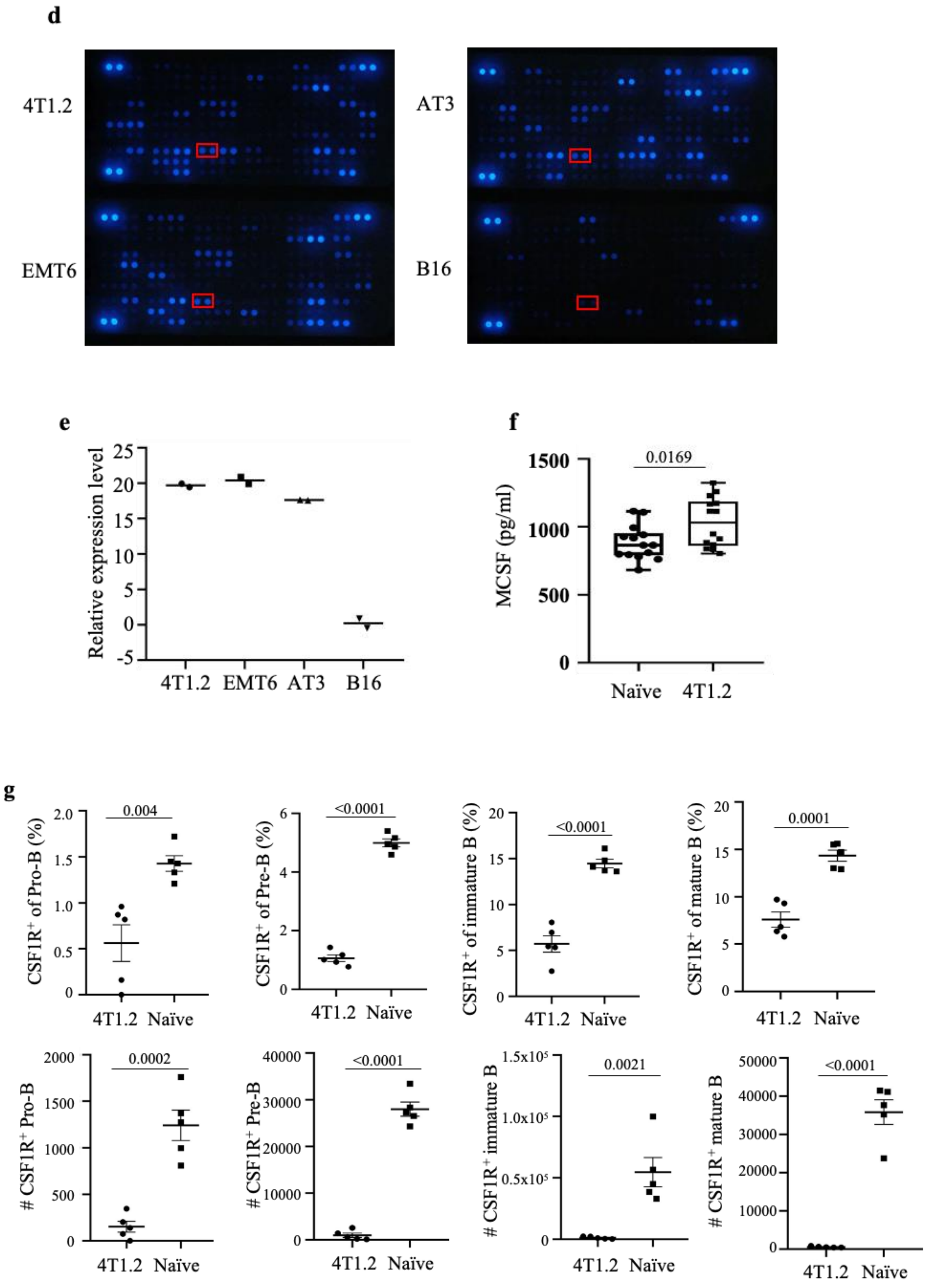
d
4T1.2
AT3
EMT6
B16
e
Relative expression level
25
20
15
10
5
0
-5
4T1.2
EMT6
AT3
B16
f
MCSF (pg/ml)
1500
1000
500
0
0.0169
Naïve
4T1.2
g
CSF1R⁺ of Pro-B (%)
0.004
4T1.2
Naïve
CSF1R⁺ of Pre-B (%)
<0.0001
CSF1R⁺ of immature B (%)
<0.0001
CSF1R⁺ of mature B (%)
0.0001
CSF1R⁺ Pro-B
0.0002
CSF1R⁺ Pre-B
<0.0001
CSF1R⁺ immature B
0.0021
CSF1R⁺ mature B
<0.0001

Suppl. Fig.7

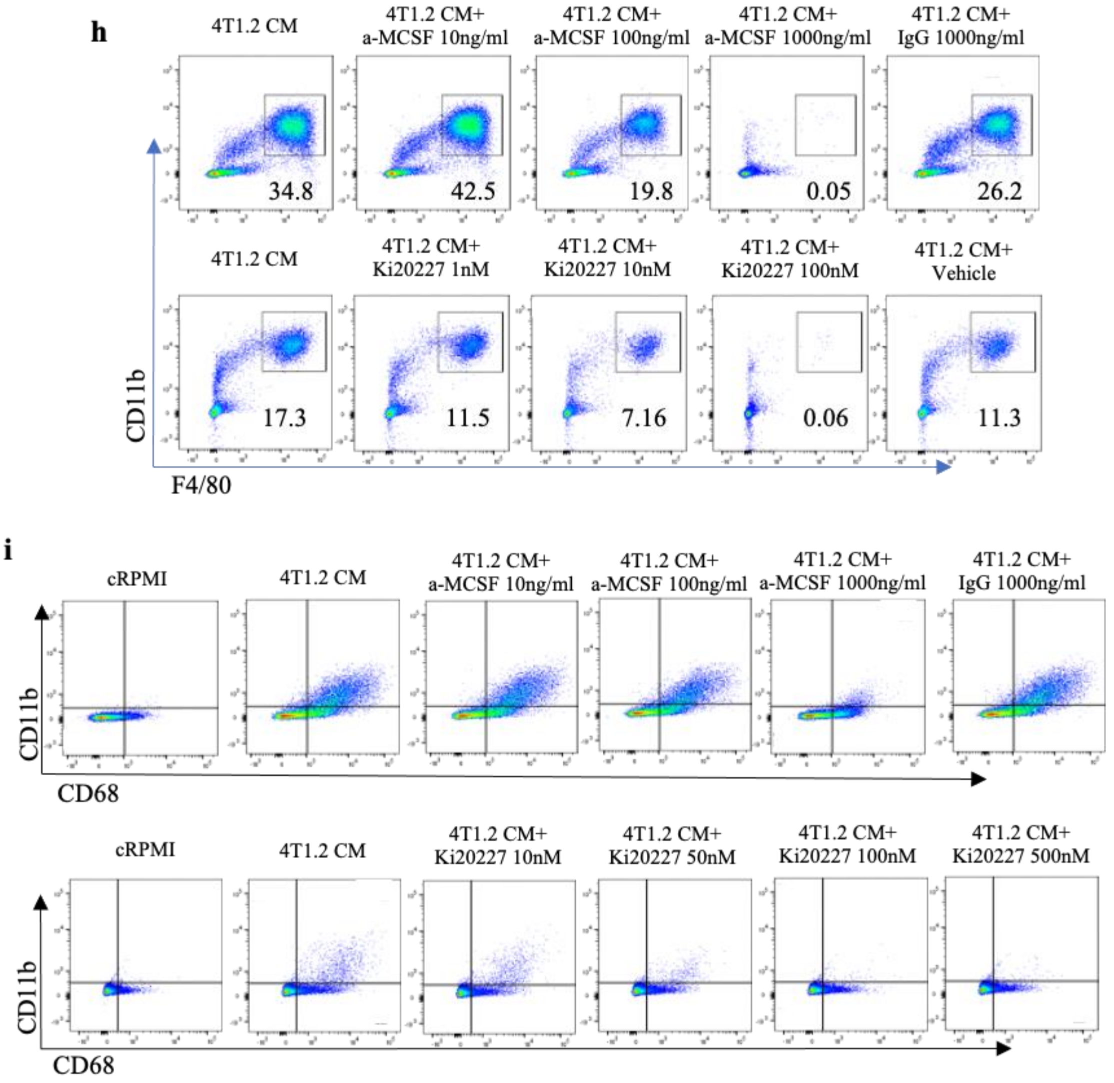

Supplementary Figure 7. CSF1/CSF1R signaling axis is needed for the generation B-MF. a, Mean ± SEM frequency (top) and numbers (bottom) of CD19$^+$B220$^+$ B cells (frequency *P*<0.0001, number *P*=0.0002) and their subsets (pro-B frequency *P*=0.0019 number *P*=0.001, pre-B number *P*<0.0001, immature B frequency *P*=0.0023 number *P*=0.0041 and mature B cells frequency *P*=0.0019 number *P*=0.001) in BM of mice with 4T1.2 cancer or naïve mice (n=5/group). *P* values were calculated using two-tailed unpaired *t* test. **b,** Numbers of B-cell precursors (CD19$^+$CD93$^+$, Mean ± SEM) in BM (*P*<0.0001), PB(*P*=0.0207), and spleen (*P*=0.0043) of mice with Mogp cancer and naïve mice (n=6 for naïve group, n=10 for Mogp group). *P* values were calculated using two-tailed unpaired *t* test. **c,** Representative FACS plots showing F4/80$^+$CD11b$^+$ cells after *in vitro* culture of splenic CD19$^+$, CD19$^+$CD93$^+$, and CD19$^+$CD93$^-$ B cells isolated from mice with 4T1.2 cancer (top) or naïve (bottom) mice in 4T1.2-CM for 7 days. **d** and **e,** Cytokine array image for expression of secreted factors in cancer CM (**D**) and relative levels (Mean) of M-CSF (red boxes in **d**) in CM of 4T1.2, EMT6, AT3 and B16-F10 cells (n=2 mouse samples per group, **e**). **f,** Box-Whiskers plot amount of MCSF (pg/ml) in sera of naïve and 4T1.2 cancer-bearing mice (n=14/group, ELISA data, *P*=0.0169, **f**).

P values in **f** were calculated using two-tailed unpaired *t* test. **g,** Mean ± SEM frequency (top) and numbers (bottom) of indicated CSF1R$^+$ B-cell subsets in BM of mice with 4T1.2 cancer or naïve mice (pro-B frequency *P*=0.004 number *P*=0.0002, pre-B frequency *P*<0.0001 number *P*<0.0001, immature B frequency *P*<0.0001 number *P*=0.0021 and mature B cells frequency *P*=0.0001 number *P*<0.0001, n=5/group). *P* values were calculated using two-tailed unpaired *t* test. **h** and **i**, anti-MCSF Ab or Ki20227 (a specific *c-fms*/CSF1R inhibitor) inhibits ability of 4T1.2-CM to generate B-MF from of BM B-cell precursors (**h**) and 70z/3 cells (**i**) in dose-dependent manner. Numbers in dot plots are for % of CD11b$^+$F/80$^+$ cells. **a-c** and **g-i** were independently reproduced at least three times.

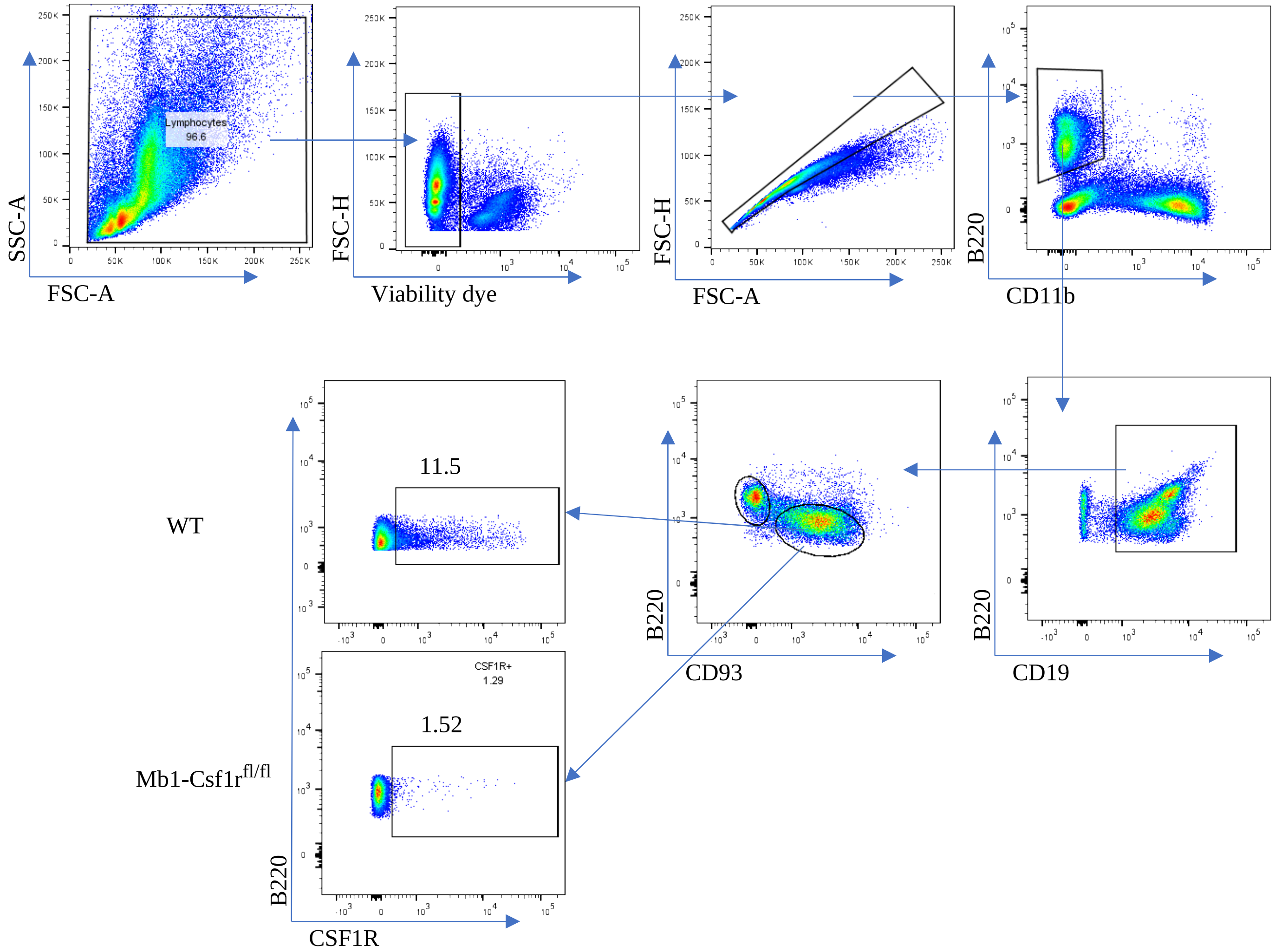

Supplementary Figure 8. Gating strategy for data presented in Fig.5F, G, showing surface CSF1R expression in BM CD93$^+$CD19$^+$ B-cell precursors of Mb1-CSF1R$^{Flox/Flox}$ (bottom) and littermate Mb1-Cre (WT, top) mice. Numbers in the pots are for expression frequency of CSF1R (%) in the gated areas.

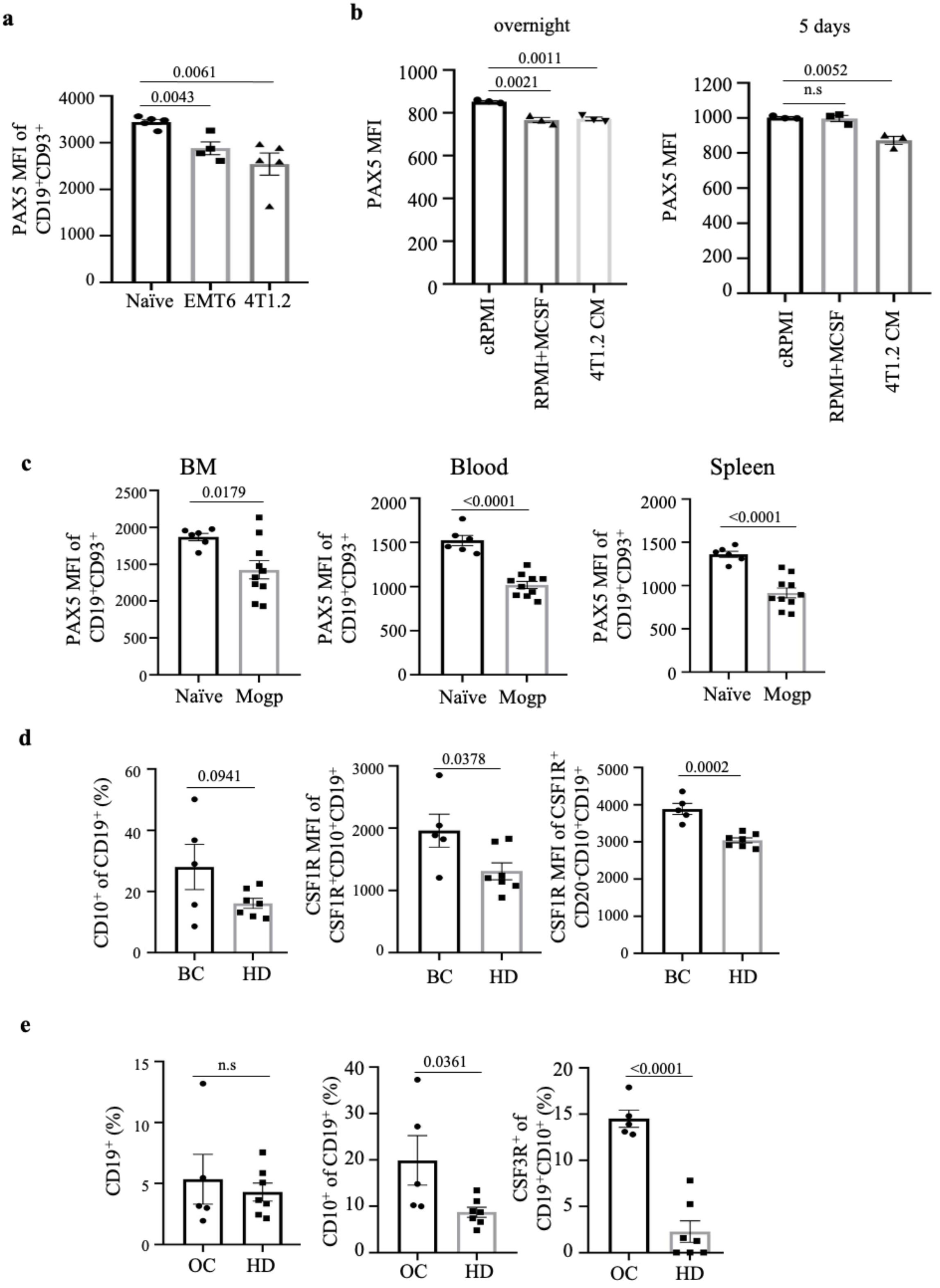

Supplementary Figure 9. Cancers induce PAX5 downregulation in B-cell precursors. a, FACS evaluation of Pax5 (MFI Mean ± SEM) in PB CD19^{+}CD93^{+} B cells from naïve mice and mice with EMT6 and 4T1.2 cancers (n=5 for naïve group, n=4 for

EMT6 group and n=5 for 4T1.2 group, *P*=0.0043 naïve vs EMT6 and *P*=0.0061 naïve vs 4T1.2). *P* values were calculated using two-tailed unpaired *t* test. **b,** Expression of Pax5 (MFI Mean ± SEM) in 70z/3 cells after treatment with M-CSF or 4T1.2-CM (n=3/group) for overnight (*P*=0.0021 cRPMI vs RPMI+MCSF and *P*=0.0011 cRPMI vs 4T1.2 CM, left) or 5 days (*P*=0.0052 cRPMI vs 4T1.2 CM, right). *P* values were calculated using two-tailed unpaired *t* test. **c,** Pax5 (MFI Mean ± SEM) in B-cell precursors ($CD19^+CD93^+$) from BM(*P*=0.0179), PB(*P*<0.0001) and spleen(*P*<0.0001) from mice with Mogp cancer and naïve mice (n=6 for naïve group, n=10 for Mogp group). *P* values were calculated using two-tailed unpaired *t* test. **d** and **e**, FACS evaluation of PB cells of breast cancer patients (n=5) and healthy human donors (n=7). *P* values were calculated using two-tailed unpaired *t* test. **d**, Mean ± SEM frequency of CD10 expression in $CD19^+$ B cells(*P*=0.0941) and CSF1R expression in $CD19^+CD10^+$ (*P*=0.0378) and $CD19^+CD10^+CD20^-$ B cells(*P*=0.0002); and **e**, $CD19^+$ cells and CD10 expression in $CD19^+$ cells(*P*=0.0361) and CSF3R expression in $CD19^+CD10^+$ cells(*P*<0.0001) in PB of ovarian cancer patients and healthy donors. *P* values were calculated using two-tailed unpaired *t* test.

Supplementary Table 1. List of antibodies used in the study

Antibody	Clone	Species reactivity	Catalog #	Company
CD19-PE	1D3	Mouse	152408	BioLegend
CD19-APC	1D3	Mouse	152410	BioLegend
CD19-BUV421	HIB19	Human	302234	BioLegend
F4/80-APC	BM8	Mouse	123116	BioLegend
F4/80-BV421	BM8	Mouse	123137	BioLegend
CD11b-BV510	M1/70	Mouse/Huma n	101263	BioLegend
CD11b-FITC	M1/70	Mouse/Huma n	101206	BioLegend
CD64-PE/Dazzle 594	X54-5/7.1	Mouse	139319	BioLegend
CX3CR1-PerCP/Cyanine5.5	SA011F11	Mouse	149009	BioLegend
CD68-BV421	FA-11	Mouse	137017	BioLegend
CD93-APC	AA4.1	Mouse	136510	BioLegend
CSF1R-PE	AFS98	Mouse	135505	BioLegend
CSF1R-PE/Cyanine7	AFS98	Mouse	135524	BioLegend
IgM-PE/Cyanine7	RMM-1	Mouse	406514	BioLegend
IgD-BV510	11-26c.2a	Mouse	405723	BioLegend
CD20-BV421	SA275A11	Mouse	150405	BioLegend
CD20-APC	2H7	Human	302310	BioLegend
CD10-PE	HI10a	Human	312204	BioLegend
CD79a-PE	F11-172	Mouse	133103	BioLegend
TER119-Biotin	TER-119	Mouse	116204	BioLegend
CD11b-Biotin	M1/70	Mouse/Huma n	101204	BioLegend
Gr-1-Biotin	RB6-8C5	Mouse	108404	BioLegend
CD3ε-Biotin	145-2C11	Mouse	100304	BioLegend
NK1.1-Biotin	PK136	Mouse	108704	BioLegend
CD49b-Biotin	HMα2	Mouse	103522	BioLegend
CD49b-Biotin	DX5	Mouse	108904	BioLegend
Ly6C-Biotin	HK1.4	Mouse	128004	BioLegend
Ly6C-FITC	HK1.5	Mouse	128005	BioLegend
Ly6G-Biotin	1A8	Mouse	127604	BioLegend
Ly6g-BV650	1A9	Mouse	127641	BioLegend
CD11c-Biotin	N418	Mouse	117304	BioLegend
CD11c-BV510	N419	Mouse	117353	BioLegend
CD117-Biotin	2B8	Mouse	105804	BioLegend

Alexa Fluor® 488 Streptavidin	-	-	405235	BioLegend
I-A/I-E-PerCP/Cyanine5.5	M5/114.15.2	Mouse	107626	BioLegend
CD206-FITC	C068C2	Mouse	141704	BioLegend
CD207-PE/Cyanine7	4C7	Mouse	144209	BioLegend
B220-FITC	RA3-6B2	Mouse/Human	103206	BioLegend
IgM-PE/Dazzle™ 594	RMM-1	Mouse	406530	BioLegend
CD93-PE	AA4.1	Mouse	136504	BioLegend
CD43-APC	S11	Mouse	143208	BioLegend
CD24-BV605	M1/69	Mouse	101827	BioLegend
CD274-PE/Cyanine7	10F.9G2	Mouse	124314	BioLegend
LAP-PerCP/Cyanine5.5	TW7-16B4	Mouse	141410	BioLegend
CD4-PerCP/Cyanine5.5	GK1.5	Mouse	100434	BioLegend
CD8a-FITC	53-6.7	Mouse	100706	BioLegend
IFN-γ-APC	XMG1.2	Mouse	505810	BioLegend
PAX5-Alexa Fluor® 488	1H9	Mouse/Human	562816	BD
CD71-BV605	C2	Mouse	563013	BD
CD124-BV421	Mil4R-M1	Mouse	564086	BD
CD366-BV421	5D12	Mouse	747626	BD
CD19-BUV395	1D3	Mouse	563557	BD
CD11b-BUV395	M1/70	Mouse/ Human	565976	BD
CD11b-BV786	M1/70	Mouse/ Human	740861	BD
CSF1R-BB515	9-4D2-1E4	Human	565346	BD
LDLR-BUV805	C7	Human	748950	BD
CSF3R-BUV395	LMM741	Human	743524	BD
Granzyme B-PE/Cyanine7	NGZB	Mouse	12-8898-82	eBioscience
FOXP3-PE	FJK-16s	Mouse	12-5773-82	eBioscience
Ki67-eFluor 450	SolA15	Mouse/Human	48-5698-82	eBioscience
CD68-PE-eFluor 610	Y1/82A	Human	61-0689-42	eBioscience
Anti-CD19 antibody[EPR23174-145]	rabbit monoclonal	Mouse	ab245235	abcam
Anti-CD68 antibody[FA-11]	Rat monoclonal	Mouse	ab53444	abcam
