# Supplementary material for: Cancer co-opts differentiation of B-cell precursors into macrophage-like cells
Source: Nat Commun. 2022 Sep 14;13:5376. doi: 10.1038/s41467-022-33117-y (PMC9474882; doi:10.1038/s41467-022-33117-y)
Supplement: Supplementary file 3 — Description of Additional Supplementary Files [file 41467_2022_33117_MOESM3_ESM.pdf]

Description of Additional Supplementary Files

File Name: Supplementary Data 1
Description: Differential expression of genes of in vitro generated B-MF (BBCM4TCM) as compared to BM B cells culture in B-cell medium. Microarray data.

File Name: Supplementary Data 2
Description: Differential expression of genes of in vitro generated B-MF (BBCM4TCM) as compared to monocyte-derived macrophages. Microarray data.

File Name: Supplementary Data 3
Description: Unique scRNA-seq transcription profile of B-MF as compared to Mo-MF.
