# Supplementary material for: Cancer co-opts differentiation of B-cell precursors into macrophage-like cells
Source: Nat Commun. 2022 Sep 14;13:5376. doi: 10.1038/s41467-022-33117-y (PMC9474882; doi:10.1038/s41467-022-33117-y)
Supplement: Supplementary file 7 — Reporting Summary [file 41467_2022_33117_MOESM7_ESM.pdf]

nature portfolio

Corresponding author(s): Biragyn, NCOMMS-21-34474B

Last updated by author(s): Jul 25, 2022

Reporting Summary

Nature Portfolio wishes to improve the reproducibility of the work that we publish. This form provides structure for consistency and transparency in reporting. For further information on Nature Portfolio policies, see our Editorial Policies and the Editorial Policy Checklist.

Statistics

For all statistical analyses, confirm that the following items are present in the figure legend, table legend, main text, or Methods section.

n/a	Confirmed	
☐	☒	The exact sample size (*n*) for each experimental group/condition, given as a discrete number and unit of measurement
☐	☒	A statement on whether measurements were taken from distinct samples or whether the same sample was measured repeatedly
☐	☒	The statistical test(s) used AND whether they are one- or two-sided<br>*Only common tests should be described solely by name; describe more complex techniques in the Methods section.*
☒	☐	A description of all covariates tested
☒	☐	A description of any assumptions or corrections, such as tests of normality and adjustment for multiple comparisons
☐	☒	A full description of the statistical parameters including central tendency (e.g. means) or other basic estimates (e.g. regression coefficient) AND variation (e.g. standard deviation) or associated estimates of uncertainty (e.g. confidence intervals)
☐	☒	For null hypothesis testing, the test statistic (e.g. *F*, *t*, *r*) with confidence intervals, effect sizes, degrees of freedom and *P* value noted<br>*Give P values as exact values whenever suitable.*
☒	☐	For Bayesian analysis, information on the choice of priors and Markov chain Monte Carlo settings
☒	☐	For hierarchical and complex designs, identification of the appropriate level for tests and full reporting of outcomes
☒	☐	Estimates of effect sizes (e.g. Cohen's *d*, Pearson's *r*), indicating how they were calculated

Our web collection on statistics for biologists contains articles on many of the points above.

Software and code

Policy information about availability of computer code

Data collection	There was no software used for data collection
Data analysis	For ATAC-seq we used https://github.com/genomicspark/ATAC-seq_QC_analysis,<br>For scRNAseq we used https://github.com/genomicspark/ESCA_Unit_Scripts

For manuscripts utilizing custom algorithms or software that are central to the research but not yet described in published literature, software must be made available to editors and reviewers. We strongly encourage code deposition in a community repository (e.g. GitHub). See the Nature Portfolio guidelines for submitting code & software for further information.

Data

Policy information about availability of data

All manuscripts must include a data availability statement. This statement should provide the following information, where applicable:

- Accession codes, unique identifiers, or web links for publicly available datasets
- A description of any restrictions on data availability
- For clinical datasets or third party data, please ensure that the statement adheres to our policy

The data associated with this study can be found in the paper or supplementary materials and ATAC-seq data are deposited at https://www.ncbi.nlm.nih.gov/geo/query/acc.cgi?acc= GSE178716 and =GSE180285

Human research participants

Policy information about studies involving human research participants and Sex and Gender in Research.

Reporting on sex and gender	All patients with either breast (n=8) or ovarian cancer (n=5) and healthy donors (n=7) were women. All donors including 13 patients participants to this research project provided written informed consent before enrollment. PBMC samples, taken before treatment were collected from all patients. Comprehensive enrollment criteria have previously been published (PMID: 29361470 and PMID: 32510664). Briefly, eligible patients had recurrent ovarian and breast cancers, an Eastern Cooperative Oncology Group (ECOG) Performance Status of 0-2 and good end-organ function.
Population characteristics	All clinical samples were PBMCs. They have recurrent breast (n=8) or ovarian cancer (n=5) and progressed on multiple prior chemotherapies prior to enrollment of clinical trial (NCT02203513).
Recruitment	All clinical samples were PBMCs not tissue samples. They have recurrent breast (n=8) or ovarian cancer (n=5) and progressed on multiple prior chemotherapies prior to enrollment of clinical trial (NCT02203513). Healthy donors (n-7) were were collected under Human Subject Protocol # 2003054 and Tissue Procurement Protocol # 2003-071.
Ethics oversight	The phase II study (NCT02203513) of prexasertib, cell cycle checkpoint kinase inhibitor, was approved by the Institutional Review Board of the Center for Cancer Research (CCR), National Cancer Institute (NCI), Bethesda, MD, USA, All patients including 13 participants to this research project provided written informed consent before enrollment and on using their samples for research. Healthy donor PBMCs (n-7) were were collected under Human Subject Protocol # 2003054 and Tissue Procurement Protocol # 2003-071 with provided written informed consent.

Note that full information on the approval of the study protocol must also be provided in the manuscript.

Field-specific reporting

Please select the one below that is the best fit for your research. If you are not sure, read the appropriate sections before making your selection.

☒ Life sciences ☐ Behavioural & social sciences ☐ Ecological, evolutionary & environmental sciences

For a reference copy of the document with all sections, see nature.com/documents/nr-reporting-summary-flat.pdf

Life sciences study design

All studies must disclose on these points even when the disclosure is negative.

Sample size	Not performed. Sample sizes of n=5-14 mice per group were considered to be sufficient as long the results were independently reproduced at least tow or three times. Sample sizes for cells were based on n=3 independent experiments repeated at least three times.
Data exclusions	None
Replication	Mouse data were independently reproduced at least twice and in vitro experiments were reproduced at least three times. With exception of experimentation's errors, most attempts were successful.
Randomization	All mice were randomized to rule out the cage effect and used the same age of female animals.
Blinding	All mice were randomized to rule out the cage effect and used the same age of female animals. Each mouse had unique ID #, and the data were analyzed in blind fashion.

Reporting for specific materials, systems and methods

We require information from authors about some types of materials, experimental systems and methods used in many studies. Here, indicate whether each material, system or method listed is relevant to your study. If you are not sure if a list item applies to your research, read the appropriate section before selecting a response.

Materials & experimental systems

n/a	Involved in the study	
☐	☒	Antibodies
☐	☒	Eukaryotic cell lines
☒	☐	Palaeontology and archaeology
☐	☒	Animals and other organisms
☒	☐	Clinical data
☒	☐	Dual use research of concern

Methods

n/a	Involved in the study	
☒	☐	ChIP-seq
☐	☒	Flow cytometry
☒	☐	MRI-based neuroimaging

Antibodies

Antibodies used	listed in the Materials and Methods and Suppl. Table 4
Validation	Validated in our previous reported studies, including Ragonnaud et al., Cancer Research, 2019; Bodogai et al., Cancer Research, 2015 and 2013.

Eukaryotic cell lines

Policy information about cell lines and Sex and Gender in Research

Cell line source(s)	Described in Methods section
Authentication	None
Mycoplasma contamination	tested for mycoplasma, all cells are free of mycoplasma. Decsribed in the Methods section
Commonly misidentified lines (See ICLAC register)	names were used as referred in literature

Animals and other research organisms

Policy information about studies involving animals; ARRIVE guidelines recommended for reporting animal research, and Sex and Gender in Research

Laboratory animals	Described in the Methods section.
Wild animals	n/a
Reporting on sex	Yes, reporeted in the Methods section
Field-collected samples	n/a
Ethics oversight	ASP 3 and the protocol approval statement is included in the Methods section

Note that full information on the approval of the study protocol must also be provided in the manuscript.

Flow Cytometry

Plots

Confirm that:

☒ The axis labels state the marker and fluorochrome used (e.g. CD4-FITC).

☒ The axis scales are clearly visible. Include numbers along axes only for bottom left plot of group (a 'group' is an analysis of identical markers).

☒ All plots are contour plots with outliers or pseudocolor plots.

☒ A numerical value for number of cells or percentage (with statistics) is provided.

Methodology

Sample preparation	Described in the Methods section
Instrument	Described in the Methods section. The results were analyzed with FlowJo v10(BD), IDEAS (Millipore) or Cytoexpert 2.3 (Beckman).

Software	The results were analyzed with FlowJo v10(BD), IDEAS (Millipore) or Cytoexpert 2.3 (Beckman).
Cell population abundance	see methods section
Gating strategy	described and shown in Suppl. Figures

☒ Tick this box to confirm that a figure exemplifying the gating strategy is provided in the Supplementary Information.
